# Driving drowsiness detection using spectral signatures of EEG-based neurophysiology

**DOI:** 10.3389/fphys.2023.1153268

**Published:** 2023-03-30

**Authors:** Saad Arif, Saba Munawar, Hashim Ali

**Affiliations:** ^1^ Department of Mechanical Engineering, HITEC University Taxila, Taxila Cantt, Pakistan; ^2^ Department of Electrical and Computer Engineering, COMSATS University Islamabad, Wah Campus, Wah Cantt, Pakistan; ^3^ Department of Computer Science, School of Engineering and Digital Sciences, Nazarbayev University, Astana, Kazakhstan

**Keywords:** electroencephalography, brain–computer interface, spectral features, drowsiness detection, feature selection, supervised learning, neurophysiology, channel selection

## Abstract

**Introduction:** Drowsy driving is a significant factor causing dire road crashes and casualties around the world. Detecting it earlier and more effectively can significantly reduce the lethal aftereffects and increase road safety. As physiological conditions originate from the human brain, so neurophysiological signatures in drowsy and alert states may be investigated for this purpose. In this preface, A passive brain-computer interface (pBCI) scheme using multichannel electroencephalography (EEG) brain signals is developed for spatially localized and accurate detection of human drowsiness during driving tasks.

**Methods:** This pBCI modality acquired electrophysiological patterns of 12 healthy subjects from the prefrontal (PFC), frontal (FC), and occipital cortices (OC) of the brain. Neurological states are recorded using six EEG channels spread over the right and left hemispheres in the PFC, FC, and OC of the sleep-deprived subjects during simulated driving tasks. In *post-hoc* analysis, spectral signatures of the *δ*, *θ*, *α*, and *β* rhythms are extracted in terms of spectral band powers and their ratios with a temporal correlation over the complete span of the experiment. Minimum redundancy maximum relevance, Chi-square, and ReliefF feature selection methods are used and aggregated with a *Z*-score based approach for global feature ranking. The extracted drowsiness attributes are classified using decision trees, discriminant analysis, logistic regression, naïve Bayes, support vector machines, *k*-nearest neighbors, and ensemble classifiers. The binary classification results are reported with confusion matrix-based performance assessment metrics.

**Results:** In inter-classifier comparison, the optimized ensemble model achieved the best results of drowsiness classification with 85.6% accuracy and precision, 89.7% recall, 87.6% F_1_-score, 80% specificity, 70.3% Matthews correlation coefficient, 70.2% Cohen’s kappa score, and 91% area under the receiver operating characteristic curve with 76-ms execution time. In inter-channel comparison, the best results were obtained at the F8 electrode position in the right FC of the brain. The significance of all the results was validated with a *p*-value of less than 0.05 using statistical hypothesis testing methods.

**Conclusions:** The proposed scheme has achieved better results for driving drowsiness detection with the accomplishment of multiple objectives. The predictor importance approach has reduced the feature extraction cost and computational complexity is minimized with the use of conventional machine learning classifiers resulting in low-cost hardware and software requirements. The channel selection approach has spatially localized the most promising brain region for drowsiness detection with only a single EEG channel (F8) which reduces the physical intrusiveness in normal driving operation. This pBCI scheme has a good potential for practical applications requiring earlier, more accurate, and less disruptive drowsiness detection using the spectral information of EEG biosignals.

## 1 Introduction

Sleep deprivation and persistent tiredness due to environmental noise and excessive traffic could be the leading cause of driving fatigue. Mental exhaustion and fatigue may cause driving drowsiness onset. Vehicle drivers are more likely to nod off at the steering wheel during long stretches of uninterrupted driving on smooth highways and straight road patches. All these reasons contribute to drowsy driving, which is a leading cause of car accidents (Ahn et al., 2016). These accidents can have devastating personal, societal, and monetary consequences, along with fatalities. So, vehicular safety and driving drowsiness detection methods to avoid dire losses are always motivational aspects for researchers.

There are several subjective and objective methods to detect drowsiness in drivers. Subjective methods include self-assessment report-based test questionnaires to measure the level of drowsiness. Karolinska Sleepiness Scale (KSS), Stanford Sleepiness Scale (SSS), Epworth Sleepiness Scale (ESS), visual analog scale (VAS), and observer-rated sleepiness (ORS) are some self-assessment approaches (Poursadeghiyan et al., 2017; Baiardi et al., 2018). These reports ask individuals to rate their tiredness by answering questions, but most subjects overestimate their drowsiness. Objective methods for driver drowsiness detection rely on measurements of the driver’s physiological and behavioral characteristics or on-road vehicle response to detect signs of drowsiness (Hu, 2017b). These methods do not rely on the driver’s self-assessment report and are considered more reliable than subjective methods. Behavioral methods incorporate computer vision algorithms that use onboard cameras to detect changes in the driver’s behavior (Akrout and Mahdi, 2021). Drowsiness is characterized by facial recognition, frequent yawning, delayed eye closures, rapid blink rates, lowered head posture, microsleep, or dozing-off behaviors (Rundo et al., 2021). However, identifying tiredness with behavioral cues, such as eye blinks, lip movement, yawn frequency, and facial features, may cause false detections. These methods are accurate for online drowsiness detection but require excessive computational power and expensive equipment to run computer vision algorithms on live video feeds (Bamidele et al., 2019). Background variation and poor ambient light might cause erroneous detections. Vehicular methods measure the drowsiness with the driving performance, which is assessed through vehicle response measured with onboard sensors. Parameters such as vehicle speed, driver’s reaction time, continuous lane deviation, missed traffic signs, and steering jerking are used to detect signs of drowsiness (Collet and Musicant, 2019). Tesla, Mercedes Benz, and others use behavioral driver assistance technologies to avoid accidents. Samsung and Eyesight collaborated to track driver attentiveness using facial patterns and features (Jabbar et al., 2020). They introduced assisted steering, automatic braking, lane departure warnings, and variable cruise control. Vehicular methods are not suitable for earlier detection as they ascertain the driver’s drowsiness when an accident is more likely. On the other hand, physiological methods measure various physiological parameters, such as eye movement with electrooculography (EOG), heart rate variability (HRV) with electrocardiography (ECG), and neurophysiological measures with electroencephalography (EEG) and functional near-infrared spectroscopy (fNIRS). These methods can detect fatigue and drowsiness using bodily organs such as the heart, muscles, eyes, and brain (Kartsch et al., 2018). Some studies additionally examine the link between drowsiness and alertness using respiratory rate, skin electrochemistry, body temperature, etc. (Adão Martins et al., 2021). These methods are disruptive to the normal driving task and potentially cause the driver discomfort but have shown promising results in detection accuracy (Arif et al., 2021b). As each method has its pros and cons, deciding between vehicular, behavioral, and physiological measures is a challenging task. Drivers require detection systems to be less intrusive as well as more accurate with earlier detection; it is a trade-off between these two aspects. However, all the bodily states primarily originated from the human brain, so it could be a potentially useful location for earlier drowsiness detection if the intrusiveness of the physiological measurement system could be reduced.

Brain activities are classified into three categories: active (intentional tasks like mental arithmetic, computation, and body motion), reactive (response to some external stimulus such as pain, audio, or video), and passive (unintentional activities like drowsiness, intelligence, possessiveness, stress, and fatigue) (Naseer et al., 2016; Qureshi et al., 2017; Nazeer et al., 2020a). Passive brain states are more difficult to detect than active and reactive brain states (Alimardani and Hiraki, 2020; Belo et al., 2021). Detection of sleep or drowsy passive states during attention-seeking tasks like driving is most crucial due to the life risks involved. Non-invasive brain–computer interfaces (BCIs) record the hemodynamic response of the brain, like changes in blood oxygen level, blood flow, and volume with fNIRS or electrophysiological signals and electrical neuronal activity with EEG. Due to low cost and effective utility, EEG and fNIRS-based BCIs are more widely used to detect activities of the brain (Arif et al., 2021b). EEG has comparatively better temporal resolution with less complex hardware, while fNIRS has good spatial resolution and more stable signals (Khan et al., 2018). Some studies are based on hybrid solutions to merge the benefits of both techniques and the application of these methods on real-life subjects (Khan et al., 2021). EEG-based passive BCI (pBCI) for drowsiness detection is a widely used method for measuring and analyzing brain activities during alertness or drowsiness (Min et al., 2017; LaRocco et al., 2020) due to its good temporal resolution. Various methods can be used to analyze the EEG signals and determine the level of drowsiness, such as frequency analysis, correlation analysis, and time domain analysis with machine learning (ML) and deep learning (DL) techniques (Aboalayon et al., 2016). DL techniques may achieve more accuracy; however, they have several potential drawbacks, including data requirements, intensive computations, black box problems, overfitting, and biases. ML algorithms are more promising and effective in avoiding such drawbacks (Khan et al., 2021).

The performance of the activity classification algorithm in the pBCI scheme mainly depends upon the number of features and their extraction complexity. Feature extraction is the process of extracting relevant and informative characteristics from the raw EEG signal to represent the drowsiness level of a driver. These characteristics, also known as features or attributes, are used as input to the classifier. There are several common feature extraction methods used in driving drowsiness detection, including temporal features extracted from the raw EEG signals in the time domain, and they include statistical measures, such as mean, standard deviation, skewness, and kurtosis (Nazeer et al., 2020b; Arif et al., 2021b; Khan et al., 2021). Frequency domain features are extracted from the EEG signals after transforming them into the frequency domain using methods such as Fourier transform, wavelet transform, or short-time Fourier transform. Examples of frequency domain or spectral features include power spectral density (PSD), alpha and beta band powers, and the ratio of different EEG frequency bands (Huang et al., 2014; Hong et al., 2018; Sasaki et al., 2019). Non-linear features are extracted using non-linear methods, such as fractal dimension, approximate entropy, and sample entropy (Li et al., 2018; Zhou and Li, 2020). These methods are used to capture the complex and non-linear dynamics of the EEG signals. However, studies have shown better classification results using spectral features with temporal correlation (Awais et al., 2017).

The channel selection approach to reduce the number of measurement electrodes is effective in reducing the intrusiveness of pBCI. EEG channel selection is the process of selecting specific electrodes on the scalp to measure brain activity from spatially localized regions. It is an important step in EEG-based drowsiness detection systems as it can affect the accuracy and reliability of the detection results. There are multiple methods for EEG channel selection, including statistical methods which use statistical significance tests to select the most informative EEG channels, such as mutual information, entropy, or variance (Alotaiby et al., 2015; Min et al., 2017). Feature-based methods use features extracted from the EEG signal, such as PSD, to select the most relevant channels with maximum information gain (Arif et al., 2021a). ML-based methods use ML algorithms to learn the relationship between the EEG signals and the drowsiness state to select the most relevant channels based on the model performance (Hu, 2017a). The commonly selected channels used in drowsiness detection include the electrodes located over the prefrontal cortex (PFC) (Fp1, Fp2), the central region of the scalp (C3, C4), frontal cortex (FC) (F7, F8), occipital cortex (OC) (O1, O2), and parietal region of the scalp (P3, P4) (Quercia et al., 2018; Kim et al., 2022). These regions are known to be associated with sleep and wakefulness and are commonly used in drowsiness detection studies (Hong and Khan, 2017; Tanveer et al., 2019).

In the preview of the aforestated introduction, this study aimed to develop an EEG-based pBCI scheme that is capable of effective drowsiness detection with less intrusion for drivers and is computationally inexpensive. For this purpose, the primary focus is on feature selection to reduce the feature extraction cost and channel selection to reduce the number of required EEG channels while obtaining higher classification results with ML-based classifiers. In this research work, raw EEG data are collected from drowsy drivers during simulated driving tasks. In a *post-hoc* analysis of neurophysiological signals, spectral features are extracted from multi-channel EEG data. Then, various feature selection approaches are applied to find the optimal features that are best representative of drowsy and alert brain states. After feature selection, extensive classification is carried out to find the single EEG channel of interest (COI), which is the significant contribution of this study. The experimental setup and adopted methodology are explained in Section 2, with detailed results presented in Section 3 and discussion in Section 4. Section 5 concludes the study with the main findings of the work.

## 2 Materials and methods

### 2.1 Participants/subjects

This experimental study was conducted on the EEG dataset collected from 12 right-handed male subjects. The average age of the recruited healthy subjects was 30 ± 2 years. All the participants had developed driving skills with experience of more than 2 years. The subjects were briefed about the study and experimental procedures before the data collection and enquired about their mental and physical fitness. None of the participants had any neurological, psychological, or mental disorders, and all had normal vision. The subjects willingly consented to participate in this study and gave written informed consent. All experiments were conducted according to the latest version of the Declaration of Helsinki. The study was reviewed and approved by the research ethics committee of HITEC University Taxila, Pakistan.

### 2.2 Experimental procedure

**FIGURE 1:**
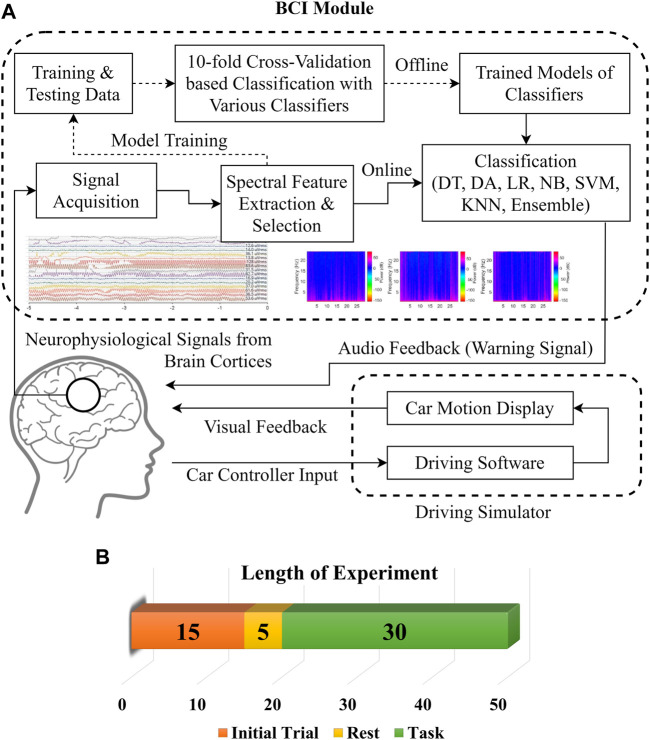
**(A)** Process flow diagram for BCI module. **(B)** Experiment details.

The experimental setup includes a driving simulator with a vehicle-like hardware controller to get a more realistic driving environment and subject responses. All the neurophysiological data acquisition experiments were conducted around 3 AM when sleep-deprived subjects felt most drowsy (Chaput et al., 2020). For all the subjects, data were recorded in multiple sessions on different days with similar environmental conditions to minimize the environmental effects on driving drowsiness. Figure 1 shows the flow diagram of the drowsiness detection scheme and details of the experiment. The subjects were required to experience and familiarize the whole experimental procedure with initial trials of 15 min at least until they got completely accustomed to the controls and environment. The later period of initial trials was used for baseline adjustment and thresholding of EEG signals subject-wise. The experiment was initiated after 5 min of rest. Subjects were assigned the lane-keeping task during simulated driving for at least 45 min, during which synchronous EEG signal recordings were performed for 30 ± 2 min. The pedestrian and traffic densities were kept at minimum in the driving environment. User display and room lighting were kept fairly dim along with quietness in the experiment room to further enhance the drowsiness-inducing conditions for the subjects. The room was vacant so that the subjects could focus on the experiment and their attention would not be diverted. The complete experiment was synchronously recorded for *post-hoc* data labeling and validation of drowsy/alert biomarkers. The subjects’ faces and physical responses were recorded with multiple cameras from different view angles and with screen recordings of driving performance. The state biomarkers were recorded for significant changes in facial expressions, eye closures, head nodding, lane deviations, abrupt physical responses, etc. The subjects were instructed to minimize unnecessary head and body motions to avoid artifacts in the neurophysiological signals.

### 2.3 Signal acquisition and processing

#### 2.3.1 Data acquisition

**FIGURE 2:**
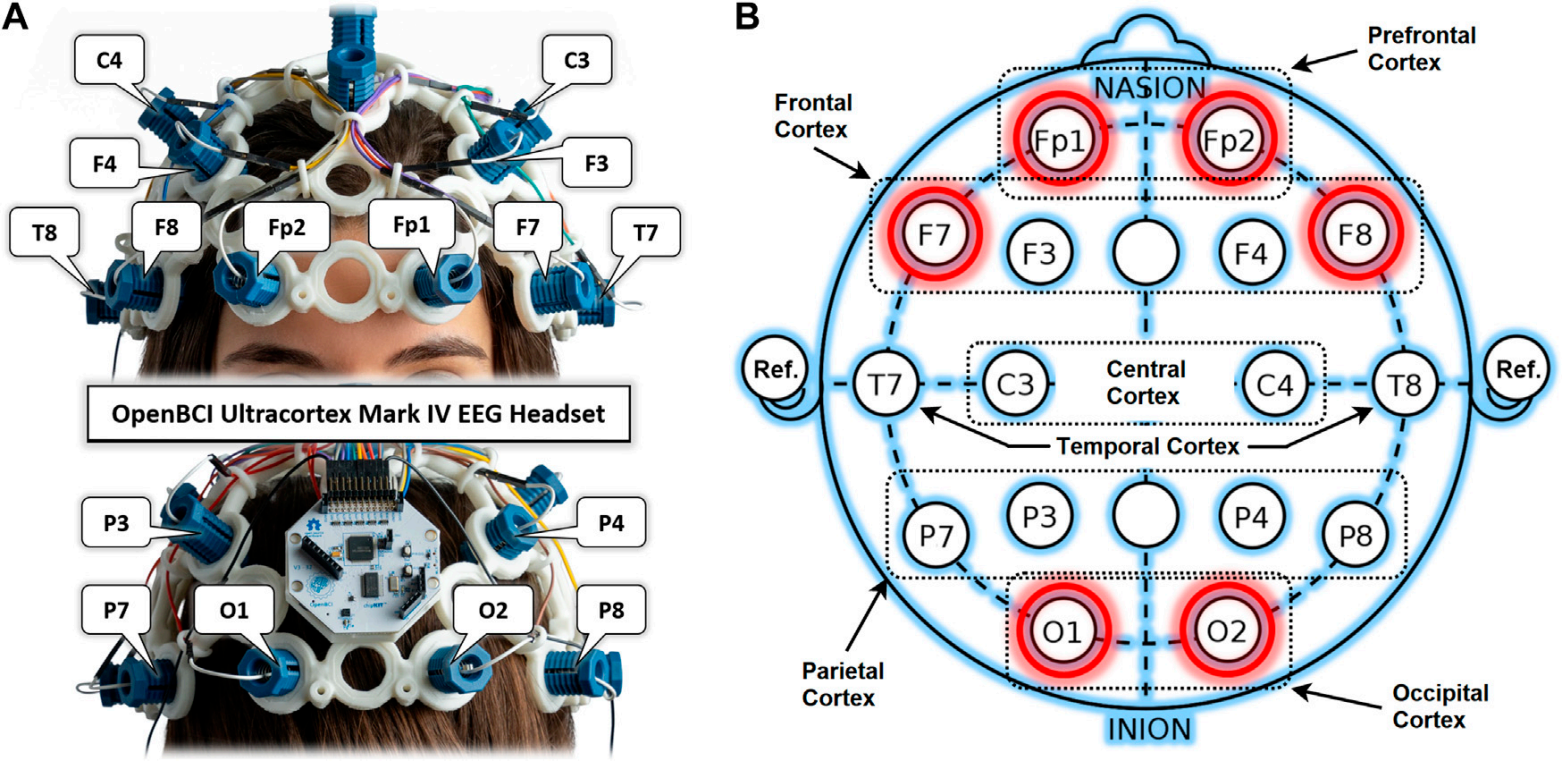
**(A)** OpenBCI Ultracortex Mark-IV EEG headset in a 16-channel configuration. **(B)** EEG referential montage according to the 10–20 system of electrode placement with selected channels highlighted in red.

An OpenBCI Ultracortex Mark-IV EEG headset in a 16-channel configuration is used to acquire neuronal activity from anterior and posterior brain regions, as shown in Figure 2A. The raw EEG signals are recorded at a 125 Hz sampling rate. Figure 2B shows the locations of 16 dry EEG electrodes placed according to the international 10–20 system. This referential montage acquires time-series data from both the right and left hemispheres of the brain in the PFC, FC, OC, temporal, central, and parietal cortices, with references set at the ear lobes. Out of these 16, only six promising channels are selected for detailed analysis based on results from previous studies. Two channels per cortex and one in each hemisphere are selected from the PFC (Fp1, Fp2), FC (F7, F8), and OC (O1, O2) for experimental dataset preparation. One of the main objectives of this study is to find the most promising brain region for drowsiness detection. A single COI is to be determined out of these six selected channels. Such localization of a single COI effectively reduces the sensory hardware, computational cost, and intrusiveness in the normal driving operation. Designed methodology and detailed analysis are performed for this localization task with promising results of drowsiness classification metrics.

#### 2.3.2 Signal processing

Signal processing with Gaussian filters was performed to remove the artifacts within certain frequency ranges and at specific frequencies. Notch reject filters were used to remove 50 Hz and 60 Hz frequencies, which are caused due to electrical interference from equipment circuits, amplifiers, and sensing boards. Artifacts due to Mayer waves and breathing were removed using band reject filters in frequency ranges of <0.01 Hz and 0.3–0.4 Hz, respectively. Higher frequency artifacts >40 Hz were removed from the data using a third-order Butterworth low-pass filter to retain the EEG frequency bands between 0.5–40 Hz. All the subjects are healthy, and none have any myogenic disorders, such as Parkinson’s disease or tremors, which indicates the absence of electromyographic artifacts in the brain signals.

### 2.4 EEG spectral signatures

#### 2.4.1 Spectral bands

The filtered frequency range of 0.5–40 Hz is further subdivided into five EEG frequency bands which are indicative of the specific physiological conditions of the human body. These are the delta ( δ : 0.5–4 Hz), theta ( θ : 4–8 Hz), alpha ( α : 8–14 Hz), beta ( β : 14–25 Hz), and gamma ( γ : 25–40 Hz) bands. δ and θ bands dominate in deep and medium sleep, respectively. α rhythms are dominant in states of light sleep, closed eyes, and relaxation (Croce et al., 2018; Liu et al., 2021), while β band is indicative of alertness, focus, and wakefulness states with cognition processes (Arif et al., 2021a). The transition from alert to drowsy state can be captured by the transition from β to α band along with the slow-wave bands ( θ and δ ) (Abidi et al., 2022; Li and Chung, 2022). The higher magnitude of spectral band powers and PSD estimates in these EEG bands for all the selected channels are analyzed to measure the patterns of alert and drowsy states of drivers.

#### 2.4.2 Power spectral density

The PSD estimates with Welch’s method for all the selected channels are computed to find the channel ranking in terms of promising representation of significant band powers over the entire band range under consideration (0.5–25 Hz). It computes the modified periodograms of overlapping segments with averaging estimation to subdue the overall spectral noise distribution and to avoid spectral information leakage. A hamming window with a 50% overlap between segments is used to calculate power distribution among frequency bins. The non-parametric spectral estimation with Welch’s PSD is computed with the following equations:

$$P_{x,m}\left(\omega_{k}\right)=\frac{1}{N{.f}_{s}}{\left|\sum_{n=0}^{N-1}x_{m}\left(n\right)w\left(n-\tau \right)e^{-\frac{i2\pi nk}{f_{s}}}\right|}^{2}$$ (1)

$${\hat{S}}_{x,ch}\left(\omega_{k}\right)=\frac{1}{K}\sum_{m=0}^{K-1}P_{x,m}\left(\omega_{k}\right)$$ (2)

where $f_{s}$ is the sampling rate, $w\left(n-\tau \right)$ is the averaging window with the overlap of τ samples, $x_{m}\left(n\right)$ is the time domain signal epoch for the m ^th^ segment of length N , $P_{x,m}\left(\omega_{k}\right)$ is the periodogram of the m ^th^ segment of signal x over $\omega_{k}$ normalized frequencies, and ${\hat{S}}_{x,ch}\left(\omega_{k}\right)$ is Welch’s PSD estimate computed by averaging the periodograms of K number of segments over the entire normalized frequency range for each selected channel ch . The PSD estimates are computed using the *pwelch* built-in function of MATLAB 9.12 (MathWorks, USA) with the aforementioned input parameters. The PSD estimates of each channel helped in establishing the inter-channel comparison in terms of spectral information distribution.

#### 2.4.3 Time-encoded spectral power

Another important spectral signature is the spectral band power synchronized with the occurrence time information. It is computed using the spectrograms which represent the time-series signals in frequency and time-frequency domains. Spectrograms computed the individual EEG band dominance in terms of spectral band power for the complete length of the experiment. They are beneficial for observing the band rhythm variations and transitions in discrete time windows synced with the driver’s state labels (Ruffini et al., 2019; Arif et al., 2021a). The spectrograms are computed using the short-time Fourier transform for all the selected channels according to the following relationship:

$$X_{ch}\left(\tau ,\omega \right)=\int_{-\infty }^{\infty }x_{ch}\left(t\right)w\left(t-\tau \right)e^{-i\omega t}dt$$ (3)

where $x_{ch}\left(t\right)$ is the time-series signal of the selected channel ch , $w\left(t-\tau \right)$ is the averaging Gaussian window function with specified overlap τ , and $X_{ch}\left(\tau ,\omega \right)$ is the time-dependent Fourier transform value over the specified time and normalized frequency resolution. The spectrograms are computed using the *pspectrum* function of MATLAB 9.12 with frequency ranges of δ , θ , α , and β bands and window time of 10 s, along with other input parameters.

### 2.5 Feature extraction

#### 2.5.1 EEG power spectrum

A total of eight spectral features are extracted from the spectral data according to the promising spectral signatures of EEG bands obtained from the spectrograms. The frequency band powers of δ , θ , α , and β bands are representative of subjects’ physiological brain states during active and passive tasks (Radüntz, 2017), so they are taken as primary features for this classification scheme. These features are extracted from the time–frequency representation of the experiment along with drowsy and alert labels by carefully synching them over time. For this purpose, the short-time power spectrum estimation is performed over the windowed segments of EEG signals for all channels over the entire duration of the experiment. A dataset is created with the mean power of each spectral band averaged out in a 10-s time window, along with weighted average-based thresholding of class labels for each observation/sample. The average spectral power of each band in each windowed segment of 10 s is calculated by the following equation:

$${\bar{P}}_{B,m,ch}=\int_{\omega_{l}}^{\omega_{h}}S_{x,m,ch}\left(\omega_{B}\right)d\omega \;s.t.\;\omega_{B}=\left[\omega_{h},\omega_{l}\right],B=\left[\delta ,\theta ,\alpha ,\beta \right]$$ (4)

where $\omega_{B}$ is the normalized frequency range of the EEG band in B with $\omega_{h}$ as the higher and $\omega_{l}$ as the lower frequency value of the band range, respectively, S is the PSD estimate of segmented EEG signal x , and $\bar{P}$ is the mean band power of the windowed segment m for each selected channel ch .

#### 2.5.2 Band power ratio

The other four spectral features are the band power ratio (BPR) indices between the four EEG bands, which are used for drowsiness detection in many studies (Ahn et al., 2016; Diaz et al., 2016). These BPR indicators are computed to capture the inter-band variations during physiological state transitions. The mostly used ratios are given as follows, which are regarded as promising predictors to classify the drowsy and alert shifts with the help of relative band power changes in each windowed segment of the experiment.

$$R_{1}=\frac{\alpha +\theta }{\beta }\;R_{2}=\frac{\alpha }{\beta }\;R_{3}=\frac{\alpha +\theta }{\alpha +\beta }\;R_{4}=\frac{\theta }{\beta }$$ (5)

These BPR indices are computed for each observation in the dataset of spectral band powers to complete the feature set. Hence, this feature set is collected for the complete length of the experiment for all the selected channels individually.

#### 2.5.3 Feature rescaling

Feature rescaling/normalization is performed when feature extraction is complete. It is performed to support the data regularization in minimizing the loss function and to achieve faster convergence during classifier model training. All the eight spectral features in the dataset are rescaled using the min-max normalization as follows:

$$X_{p,ch}^{\prime }=a+\frac{X_{p,ch}-\min\left(X_{p,ch}\right)}{\max\left(X_{p,ch}\right)-\min\left(X_{p,ch}\right)}\left(b-a\right)$$ (6)

where $X_{p,ch}$ is the extracted value of the feature p in the dataset of the channel ch , $X_{p,ch}^{\prime }$ is the rescaled or normalized value of the feature which will be supplied to the classifier for training, b is the upper and a is the lower limit of the normalization range, respectively, which is defined as $\left[\begin{array}{cc} a & b \end{array}\right]=\left[\begin{array}{cc} 0 & 1 \end{array}\right]$ for all the features in the dataset.

**FIGURE 3:**
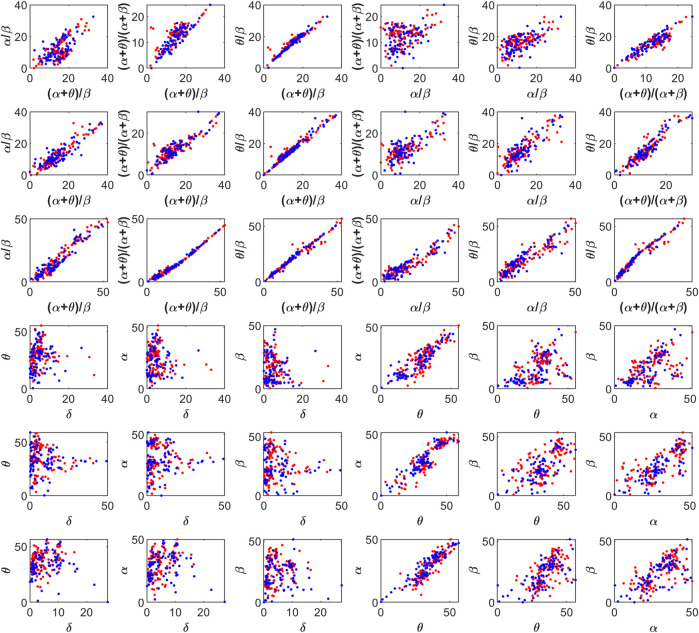
2D scatter plots on a log scale for feature pairs between band power ratios (top three rows), and spectral band powers (bottom three rows). Fp1, Fp2, O1, O2, F7, and F8 channels in each row from top to bottom, respectively. Red and blue data points represent drowsy and alert states, respectively.

**FIGURE 4:**
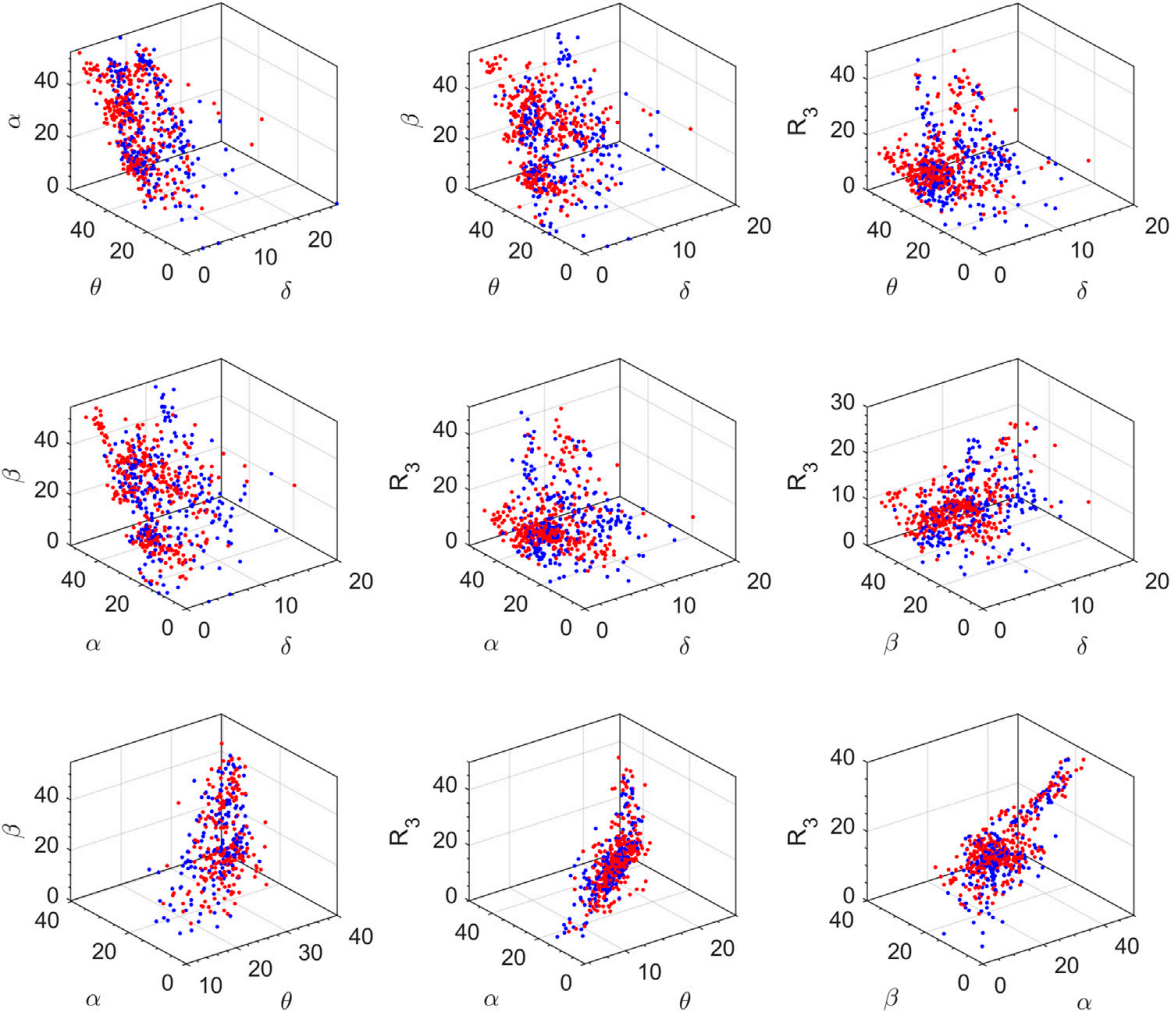
3D scatter plots on a log scale for all the ternary combinations of five top-ranked features. Red and blue data points represent drowsy and alert states, respectively (channel F8, all subjects).

The feature space for this BCI scheme comprises all the possible two- to five-dimensional feature combinations of all the spectral features. The combination of all eight features is also included in the feature space. The complete feature space is used for extensive experimentation to find the optimal combination of features, resulting in the best accuracy of drowsiness detection. Figure 3 shows the scatter plots for promising 2D feature pairs of spectral band powers and BPR indices in selected channels. From top to bottom, each row shows the plots for Fp1, Fp2, O1, O2, F7, and F8 channels, respectively. Figure 4 shows the 3D scatter plots for all the possible ternary combinations of five selected features which resulted from the global feature selection approach, as described in the next section. The red and blue data samples from drowsy and alert classes, respectively, are plotted against the respective band power magnitudes on the axes in these feature space plots. The feature values are displayed over a logarithmic scale for better visualization of class dispersion. These plots are analyzed to visually observe the data dispersion and assess the degree of non-linearity involved in class separation boundaries between drowsy and alert states.

### 2.6 Feature selection approaches

Multiple feature selection methods are used in this study to find the most representative and optimum number of features which give the best prediction results. Feature selection approaches are used to aid the reduction in data dimensionality and computational costs. The classifier training process becomes faster, and convergence is achieved earlier with the optimum number of features used. Primarily, three filter-type feature ranking approaches are applied to this spectral EEG feature set. The embedded-type feature selection approach is also used by training multiple classifiers with developed feature space. The primary feature ranking approaches are discussed in the following section.

#### 2.6.1 Minimum redundancy maximum relevance method

The minimum redundancy maximum relevance (MRMR) feature selection method ranks all the features in the feature set in order of maximum inter-feature dissimilarity to subdue the redundant features. Meanwhile, it also checks the maximum relevance of ranked features with the target variable. It ranks the features with a balanced ratio of both these aspects to end up with an optimized feature set ordered by descending predictor importance score (Fathima and Kore, 2021; Pudjihartono et al., 2022). For this purpose, this algorithm performs the redundancy check on all the possible 2D feature pairs of the feature space. The entropy-based mutual information index $I\left(p_{i},p_{j}\right)$ is computed for each feature pair with $p_{i}$ and $p_{j}$ as mutual predictors under consideration. Based on these values of all the pairs, the objective is to find such an optimal feature set F which has minimum redundancy value $W_{F}$ , as described by the following equation:

$$\min\;W_{F}=\min\left(\frac{1}{{\left|F\right|}^{2}}\sum_{p_{i},p_{j}\in F}I\left(p_{i},p_{j}\right)\right)$$ (7)

Furthermore, the algorithm also performs the relevance check between all the features and responses in the dataset. The obtained optimal feature set F must have maximum mutual relevance value $V_{F}$ of predictors $p_{i}$ and response variable y based on their entropy-based mutual information index $I\left(p_{i},y\right)$ , as shown in the following equation:

$$\max\;V_{F}=\max\left(\frac{1}{\left|F\right|}\sum_{p_{i}\in F}I\left(p_{i},y\right)\right)$$ (8)

The final predictor importance score is computed by maximizing the mutual information balance for each feature which is the ratio of feature relevance to the feature redundancy. The MRMR method-based predictor importance scores are computed using the *fscmrmr* built-in function of MATLAB 9.12.

#### 2.6.2 Chi-square (*χ* ^2^) test

The $\chi^{2}$ test is the statistical analysis-based univariate feature importance calculation method. It computes the predictor importance scores based upon the p -values of the statistical dependence test between each predictor and the response variable. p -values less than the alpha value of 0.05 effectively reject the null hypothesis, indicating that those predictors are important to classify this response variable (Khan et al., 2020). A negative logarithmic function is applied to the achieved p -value of each test, which gives the feature importance score for feature ranking. The $\chi^{2}$ statistics are calculated with the following formula:

$$\chi_{p}^{2}=\sum_{i=1}^{k}\frac{{\left(O_{i}-E_{i}\right)}^{2}}{E_{i}}$$ (9)

where $O_{i}$ and $E_{i}$ are the observed and expected frequency distributions, respectively, of the i ^th^ pair between the discrete bin of predictor p and a single class of the response variable y , and k is the total number of such combinations between the 10 discretized bins of the continuous predictor and two classes of the response variable. The chi-squared statistics are calculated for each feature, and the features which effectively reject the null hypothesis of feature and response dependency, with a 95% confidence interval, are taken as important features. These important features are then ranked based on the magnitude of their test statistics scores. The $\chi^{2}$ test-based feature selection is implemented using the *fscchi2* built-in function of MATLAB 9.12.

#### 2.6.3 ReliefF algorithm

This filter-type feature selection method measures the quality of features in terms of their ability to effectively differentiate the neighboring samples when they belong to different classes. Unlike other feature selection approaches which measure the dependence of features and the response variable, this method determines the feature importance by examining the number of observations. This algorithm increases or decreases the weight of each feature based on the comparative variance in its value for the same and different class neighboring samples (Fathima and Kore, 2021; Pudjihartono et al., 2022). All feature weights are initialized with zero and updated after each iteration of the algorithm. In each iteration, the algorithm selects a random observation and finds the *k* number of nearest neighboring observations from each class of the response variable using the Manhattan distance metric or L1 norm. After neighbor selection, the feature vectors of all the observations are compared feature-wise, and the cumulative magnitude of difference in each predictor’s value is computed. If a feature shows less variance for observations in the same class as compared to those from different classes, then its weight is updated with an increase. The weight update of the feature is penalized with a decrease if its value shows comparatively higher variance for the neighboring observations of the same class. Similarly, the weights of all the features get updated at each iteration of the algorithm. The following relationships represent the feature weight updating criteria of the algorithm:

W∀fi=W∀fi−1−∑j=1kΔ∀fri,hj,ptk+∑∀nPn1−Pp∑j=1kΔ∀fri,mj,ntk (10)

$$\Delta_{\forall f}\left(r_{i},h_{j,p}|m_{j,n}\right)=\frac{\left\|f_{r}-f_{h}|f_{m}\right\|}{\max\left(f\right)-\min\left(f\right)}\;s.t.\;i=1,2,\ldots ,t,f\in F$$ (11)

where W∀fi is the updated weight of the feature f in the i ^th^ iteration of the algorithm and is being computed for all the features of the complete feature vector F , k is the total number of nearest neighboring observations set as 10 here, t is the total number of ReliefF iterations, $r_{i}$ is the randomly sampled observation with the nearest neighbor $h_{j,p}$ , and both belong to the same class p having $P\left(p\right)$ occurrence probability, $m_{j,n}$ are the nearest neighbors of $r_{i}$ but belong to different class n having $P\left(n\right)$ probability of occurrence in the dataset, $\Delta_{\forall f}\left(r_{i},h_{j,p}|m_{j,n}\right)$ is the difference in feature value of observation r with the same class neighbor h and with the different class neighbor m .

The ReliefF algorithm performs such iterations for all the observations to increase its reliability with the highest probability estimates, particularly for smaller datasets having a lesser number of observations. This exhaustive computation is well suited for smaller datasets but incurs computational penalties with larger datasets (Pudjihartono et al., 2022). In that case, the choice of random sampling for observations is best with the significant number of iterations by keeping in view the computational cost admissibility. Generally, the number of algorithm iterations is dependent upon the probability estimates of the required reliability level. Finally, the features are ranked according to their weights after complete iterations of the algorithm. The predictor importance score is calculated with this algorithm using the *relieff* built-in function of MATLAB 9.12.

#### 2.6.4 Global predictor importance

The global predictor importance (GPI) is calculated to find the global feature ranking influenced by each of the aforementioned feature selection methods. As all three approaches give predictor importance scores in different ranges with different means and variances, they cannot be averaged out to get their cumulative effect. Normalizing the importance scores in the same range with typical normalization methods and calculating their mean is not a viable solution either because this approach results in altering the skewness and kurtosis of the original shape distribution (Nazeer et al., 2020b; Singh and Singh, 2020). The considerable drop in the feature importance score of adjacent ranked features is a considerable factor in feature selection as it shows the decrease in response predictive power of low-ranked features. Skewness and kurtosis of the original distribution must be retained when getting the cumulative effect. For this purpose, the GPI is calculated as follows, using the Ζ -score-based standardization of feature importance scores to gauge all the results on the same basis and then averaging out to get the GPI:

$$GPI=\frac{1}{3}\sum_{i=1}^{3}{Ζ}_{\forall f,i}=\frac{1}{3}\sum_{i=1}^{3}\left(\frac{x_{\forall f,i}-\mu_{\forall f,i}}{\sigma_{\forall f,i}}\right)$$ (12)

where $x_{f,i}$ is the feature importance score of a feature f in the i ^th^ feature selection method, μ and σ are the mean and standard deviation of the feature importance score, respectively, and Ζ is the Ζ -score-based standardized value of the feature importance score. In this way, all the feature scores of all schemes are represented with zero mean value and score spread in the same standard deviation bounds. The Ζ -score standardization is performed in MATLAB 9.12 using its built-in function *normalize*. All the feature selection methods are applied to the channel-wise combined EEG data of all the subjects.

### 2.7 Classification

Multiple supervised learning-based classification methods are used to perform binary classification of drowsiness and alert neurophysiological states of driving subjects. Seven classifiers are used in this study: decision trees (DT), discriminant analysis (DA), logistic regression (LR), naïve Bayes (NB), support vector machines (SVM), *k*-nearest neighbor ( k NN), and ensembles. All the classifiers are trained, tested, and validated on a complete feature set and a GPI-based selected feature set. In addition, 10-fold cross-validation is incorporated in all training sessions to overcome the overfitting issues and maintain the generalization in predictions of the classifiers. An exhaustive approach is used for activity classification to achieve optimum results using various variants of all the classifiers and multiple feature sets. An analysis is performed independently for all the selected EEG channels to find the most promising brain region in terms of COI to achieve the best results for drowsiness detection. Furthermore, the hyperparameters of the best-performing classifier are optimized with the Bayesian optimization technique to achieve optimum performance of classification results.

#### 2.7 1 Decision trees

The classification DT algorithm expands the flowchart-like tree structure with features at branches, feature split tests at nodes, and classification decisions at leaf nodes. The tree data structure expands to include features with minimum entropy and maximum information gain, leading to zero entropy leaf nodes which are class labels. This classifier is implemented using the *fitctree* built-in function of MATLAB with three sets of hyperparameters. The tuned hyperparameter is the maximum number of splits which is the tree depth controlling factor, while the other hyperparameter is the split criterion which is fixed to Gini’s diversity index. The tree depth controlling factor is a significant parameter that should be increased to increase the classification accuracy at the expense of increased training time. The increase in the predictive power of the DT also increases its complexity level. Highly blended data dispersion of distinct classes requires a greater number of tree splits to draw fine class-distinction boundaries. The increase in non-linearity of class separation boundaries requires a greater number of splits to achieve better classification accuracy. Three presets of DT are tested here with 100, 20, and 4 splits at maximum and are known as fine, medium, and coarse trees, respectively.

#### 2.7.2 Discriminant analysis

The DA classifier tends to establish a linear or quadratic combination of features to make simpler decision boundaries between different class data. Generally, it has a linear and a quadratic variant, known as linear discriminant analysis (LDA) and quadratic discriminant analysis (QDA), respectively, along with diagonal linear and diagonal quadratic variants. LDA draws linear class separation boundaries with the assumption of Gaussian distribution of the classes, while QDA draws non-linear class separation boundaries of parabolic, hyperbolic, or elliptical trends for blended data dispersion. These methods use the full covariance structure of the data and converge rapidly. They have good classification accuracy for distinctive data scatters. Both variants are implemented using the *fitcdisc* function of MATLAB.

#### 2.7.3 Logistic regression

The LR classifier uses the logistic or sigmoid function to classify the class probabilities for binary classification problems. The binomial LR classifier is implemented here using the generalized linear regression model *fitglm* function of MATLAB. Like DA methods, LR is also faster to train, but classification accuracy may degrade when data scatter plots do not have distinctive class separation boundaries. The binomial logistic regression function is represented by the following equation:

$$p\left(x\right)=\frac{e^{\left(a_{1}x+a_{0}\right)}}{1+e^{\left(a_{1}x+a_{0}\right)}}$$ (13)

where x is the input feature value, $a_{1}$ and $a_{0}$ are the binomial coefficients, and $p\left(x\right)$ is the probability of the feature being mapped to either of the binary classes of the response variable.

#### 2.7.4 Naïve Bayes classifier

The NB classifier performs probabilistic classification based on Bayes’ theorem with an assumption of inter-feature independence. It works on prior and posterior probabilities of features with probability density estimation functions. The two presets for numeric predictors are the Gaussian NB and kernel NB which are applied here using the *fitcnb* function of MATLAB. The Gaussian NB works on the assumption of normal distribution of the data among response classes. The kernel NB applies the kernel distribution function on numeric predictors with Gaussian-type kernel smoother. The NB classifiers are generally faster in training convergence but achieve good classification accuracy with the categorical predictors as compared to numeric features.

#### 2.7.5 Support vector machines

The SVM classifier establishes the hyperplanes for class separation boundaries using the polynomial kernel function and radial basis function (RBF) kernels. The objective of the algorithm is to find the hyperplane with the maximum margin. The linear, quadratic, and cubic polynomial kernel functions are used to generate respective hyperplanes, but they are computationally expensive for higher dimensional data and take a significant amount of training time without a significant increase in accuracy (Qureshi et al., 2016). On the other hand, the RBF kernel-based Gaussian SVM achieves better classification accuracies with less training time for higher dimensional data as well. They are also effective for classifying the non-linear boundaries of mixed-class data dispersions. The SVM is applied using the *fitcsvm* function of MATLAB for this binary classification problem. Three Gaussian SVM presets are used here, namely, fine, medium, and coarse Gaussian SVM, which differ by Gaussian kernel scale of values $\frac{\sqrt{P}}{4}$ , $\sqrt{P}$ , and $\sqrt{P}\times 4$ , respectively, where P is the number of features. The hyperparameter of respective kernel scale values is 0.71, 2.8, and 11 for the drowsiness detection scheme with eight features. The SVM variants accordingly create fine, medium, and coarser distinctions and separation hyperplanes between detection classes. Fine Gaussian SVM is preferred over other variants with highly non-linear and blended data dispersion of different classes. The other hyperparameter is the box constraint parameter level C , which is set to 1 to allow the flexible soft margin penalty due to highly non-linear data dispersion.

#### 2.7.6 k -nearest neighbor

The k NN algorithm classifies the data points in respective classes based on the classes of their k -nearest neighbors found with different distance metrics. This classifier is implemented using the *fitcknn* function of MATLAB with its presets. The available hypermeters are the number of nearest neighbors, distance metrics used to find nearest neighbors, and distance weights which make multiple presets with different combinations. The fine, medium, and coarse k NN made fine, mid-level, and coarser distinctions and class separation boundaries with 1, 10, and 100 numbers of nearest neighbors, respectively, while classifying the neighboring new data points in the neighborhood of their respective classes. These three presets use the Euclidean distance metric with unbiased and equal-distance weighting function, with the only difference being the number of nearest neighbors. The other three presets—cosine, cubic, and weighted k NN—are the subtypes of medium k NN (10 number of nearest neighbors) with the difference of distance metric and distance weighting functions. Cosine and cubic k NN use cosine and Minkowski (cubic) distance metrics, respectively, with no distance weighting function or equal weights. Weighted k NN is the same as medium k NN, with the only difference in biased distance weights. Distance-weighting functions used in weighted k NN are inverse ( $\frac{1}{D}$ weight) or squared inverse ( $\frac{1}{D^{2}}$ weight), where D is the distance between neighboring data points. Among all these presets, weighted k NN is more likely to outperform due to highly non-linear data dispersion between feature pairs of both the EEG feature spaces. k NN algorithms are among the classifiers with higher classification accuracy, relatively faster convergence, and less training time.

#### 2.7.7 Ensemble classifier

The ensemble classifier aggregates the cumulative performance of various weak learners to achieve higher accuracy. It is implemented using the *fitcensemble* function of MATLAB with various hyperparameters like the ensemble method, the maximum number of splits for DT learners, the number of weak learners, and the learning rate. The number of learners is set to 30 to avoid delayed convergence and yet achieve higher detection accuracy. The adaptive boosting (AdaBoost) and random under-sampling boost (RUSBoost) ensemble methods with a learning rate of 0.1 and 20 maximum splits are the boosted trees and RUSBoosted trees, respectively. The bootstrap aggregating (bagging) ensemble method with random forest DT and 3002 number of splits is bagged trees. The subspace discriminant (DA learner) and subspace k NN ( k NN learner) ensemble methods use randomly selected 4D subspace of features in datasets with a large number of predictors. The bagged and boosted trees tend to achieve higher accuracies for datasets with a smaller number of predictors due to fine DT learners performing well in non-linear class separation boundaries and blended data dispersion (Ali et al., 2023).

### 2.8 Performance evaluation metrics

The confusion matrix-based classification performance evaluation metrics are used to show the results of this study. In this driving drowsiness detection scheme, the drowsy brain state is a positive class ( P ) with the class label “1,” and alert is the negative class ( N ) with the class label “0.” True positive ( TP ) is the count of instances in which the actual drowsy state or positive class is correctly predicted as positive, while true negative ( TN ) is the count of correct predictions of alert state or negative class. False negative ( FN ) is the count of observations in which the drowsy subject is wrongly identified as alert or N , while false positive ( FP ) is the count of alert states which are incorrectly predicted as drowsy or P . Based on this set of observations, the following performance evaluation metrics are calculated for this binary classification problem.

Classification accuracy gives the measure of the correct prediction power of the classifier with the ratio of correct prediction of both the brain states to the total population of the dataset. It is calculated as

$$Accuracy=\frac{TP+TN}{TP+FN+FP+TN}$$ (14)

Precision or positive predictive value ( PPV ) measures the effective drowsiness prediction capability of the classifier with the ratio of accurate drowsy predictions to the total number of drowsy predictions.

$$Precision,PPV=\frac{TP}{TP+FP}$$ (15)

Recall, sensitivity, or true-positive rate ( TPR ) is the measure of probability or percentage of correct drowsiness detections out of all the observations which are labeled as drowsy in the dataset.

$$Recall,Sensitivity,TPR=\frac{TP}{TP+FN}$$ (16)

F_1_-score gives the harmonic mean of the sensitivity and precision of the classifier to find a balanced measure of correct identification of drowsiness out of all the positive predictions and all actual positive states in the dataset.

$$F_{1}\;score=\frac{2\times TP}{2\times TP+FP+FN}$$ (17)

Specificity, selectivity, or true-negative rate ( TNR ) is the capability of the classifier to accurately identify the alert states which gives the performance measure of the classifier to correctly reject the negative class. It ascertains the balanced efficiency of the trained model with an increased confidence level of classification results.

$$Specificity,Selectivity,TNR=\frac{TN}{FP+TN}$$ (18)

Matthews correlation coefficient (MCC) represents the relationship between the actual and predicted responses in the presence of a class imbalance in the dataset (Akhtar et al., 2022). It overcomes the bias effect in the predictions caused by the probability shift due to class imbalance and gives a reliable and balanced measure of the classifier’s prediction capability.

$$MCC=\frac{\left(TP\times TN\right)-\left(FP\times FN\right)}{\sqrt{\left(TP+FP\right)\left(TP+FN\right)\left(TN+FP\right)\left(TN+FN\right)}}$$ (19)

Cohen’s kappa coefficient ( κ ) is the statistical test to measure the degree of reliability or agreement between two raters (Akhtar et al., 2021). The kappa score κ≈1 shows that both the performance evaluation raters are in complete agreement about their performance, and their results are reliable, while κ≈0 indicates the disagreement in raters and such results happen by chance (Ali et al., 2023).

$$\kappa =\frac{2\times \left(TP\times TN-FP\times FN\right)}{\left(TP+FP\right)\times \left(TN+FP\right)+\left(TP+FN\right)\times \left(TN+FN\right)}$$ (20)

Fall-out or false-positive rate ( FPR ) shows the probability of false detection, which is the ratio of those alert states which are wrongly predicted as drowsy to the total number of alert state observations.

$$Fall\;out,FPR=1-TNR=\frac{FP}{TN+FP}$$ (21)

In addition to the aforementioned metrics, the overall performance of the trained classifiers is shown by the receiver operating characteristics (ROC) curve and area under the curve (AUC), which is plotted between the recall and fall-out. The well-trained models have higher TPR and lower FPR with AUC near 1.

## 3 Results

### 3.1 EEG spectral analysis

**FIGURE 5:**
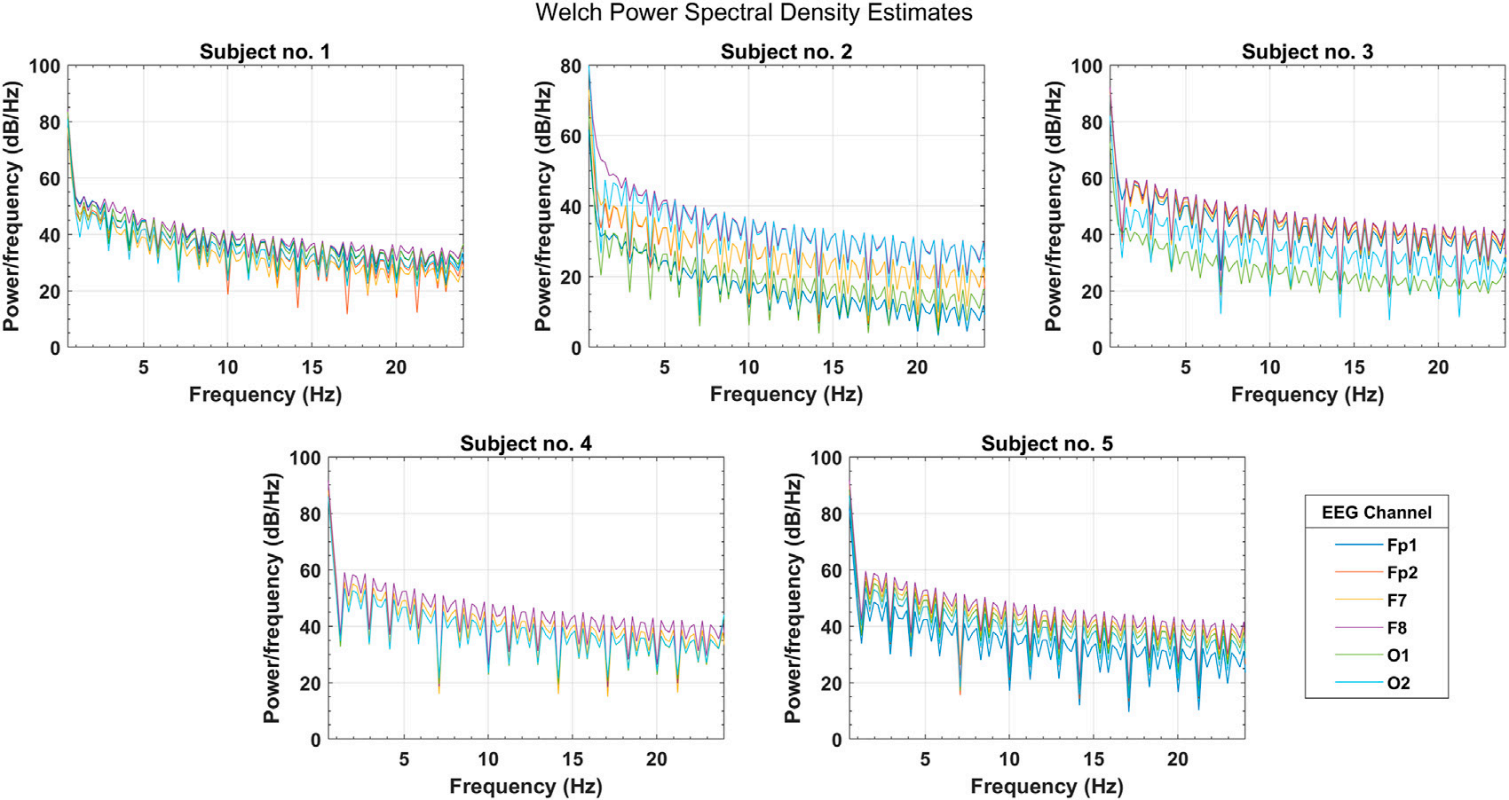
Welch’s power spectral density estimates for selected PFC, FC, and OC channels over the spectral range of four EEG bands (subjects 1–5).

In the initial testing of the data, Welch’s PSD estimates were analyzed to observe the spectral information gain among selected EEG channels. Figure 5 shows the PSD distribution over the complete frequency range of δ,θ,α,β EEG bands in the selected PFC, FC, and OC channels. The magnitude of spectral power distribution shows which channels contain comparatively more spectral density so that they could be targeted for spectral feature extraction and activity classification. In inter-cortex comparison, the FC (F7, F8) contains comparatively more power in each frequency band ranging from 40 to 80 dB/Hz for most of the subjects. For other selected channels, this range varies among many subjects and drops to 10 dB/Hz in some cases. In lateral brain regions, the right hemisphere is comparatively more dominant, with Fp2, F8, and O2 having higher power magnitudes in all the bands as compared to their left hemisphere counterparts. This observation indicates the reliability of FC for spectral analysis.

**FIGURE 6:**
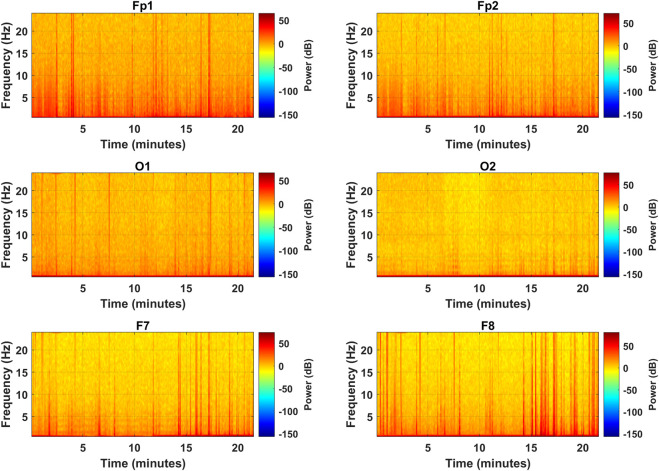
Spectrograms for the complete experiment over the frequency range of all EEG bands for the selected channels (subject 2).

After the PSD estimates, the spectrograms were visually analyzed to access the effectiveness of the selected channels in representing the frequency distribution among the EEG bands. Figure 6 represents the spectrograms computed for each selected channel over the complete length of the experiment showing the spectral band power distribution. The vertical orange spikes rising above 20 Hz are the time instances of alert states when subjects were engaged in mental computation during the driving task. In such scenarios, band powers are higher among all the bands, especially in the β band, as can be seen more clearly in PFC and FC. In drowsy instances and eye closures, higher band power can be observed in the α band, as shown in O2 at about 10 Hz, and in the δ band at various individual frequencies in O2 and F7 (Arif et al., 2021a). Here, the F8 channel is the most promising in distinguishing intermittent brain states as it shows more crisp and differentiable peaks of alert states from the drowsy periods. Inter-band power variations can be seen more distinctively in this channel, and it distinguished relatively more alert instances among all. This spectral analysis declares the F8 channel as the best candidate for COI.

**FIGURE 7:**
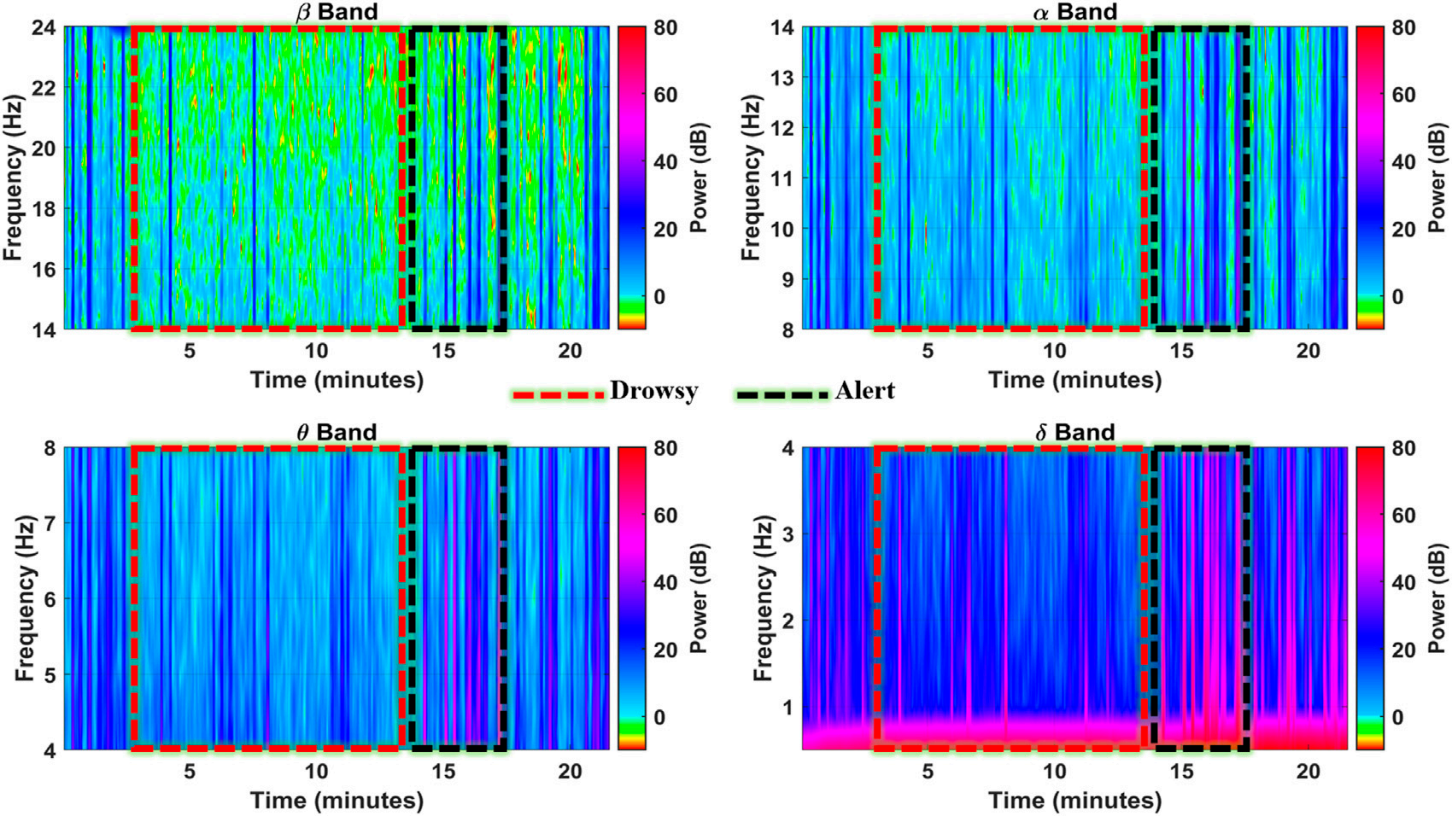
Spectrograms showing spectral band powers in individual EEG frequency bands in the F8 channel (subject 4).

The F8 channel was further investigated band-wise to ascertain the individual band powers as representative features for brain state classification. The subject was alert at the start and end of the experiment, with intermittent alert states at the 16th, 17th, and 19th minutes, as shown in Figure 7. During these instances, the higher band power spikes in all the bands can be observed with up to 35 dB, 40 dB, 45 dB, and 50 dB magnitudes in β , α , θ , and δ bands, respectively. There is a significant loss in β band power magnitude in drowsy periods, while total band power magnitude is only contained in the drowsiness-related bands (Cui et al., 2022). Even the α band power significantly drops in more drowsy instances like the 9th and 10th minutes, with all the power contained only in the θ and δ bands (Awais et al., 2017). These inter-band power variations with changing neurophysiological states support the confidence in using spectral band power and BPR indices as features for drowsiness classification.

### 3.2 Feature selection scores

**FIGURE 8:**
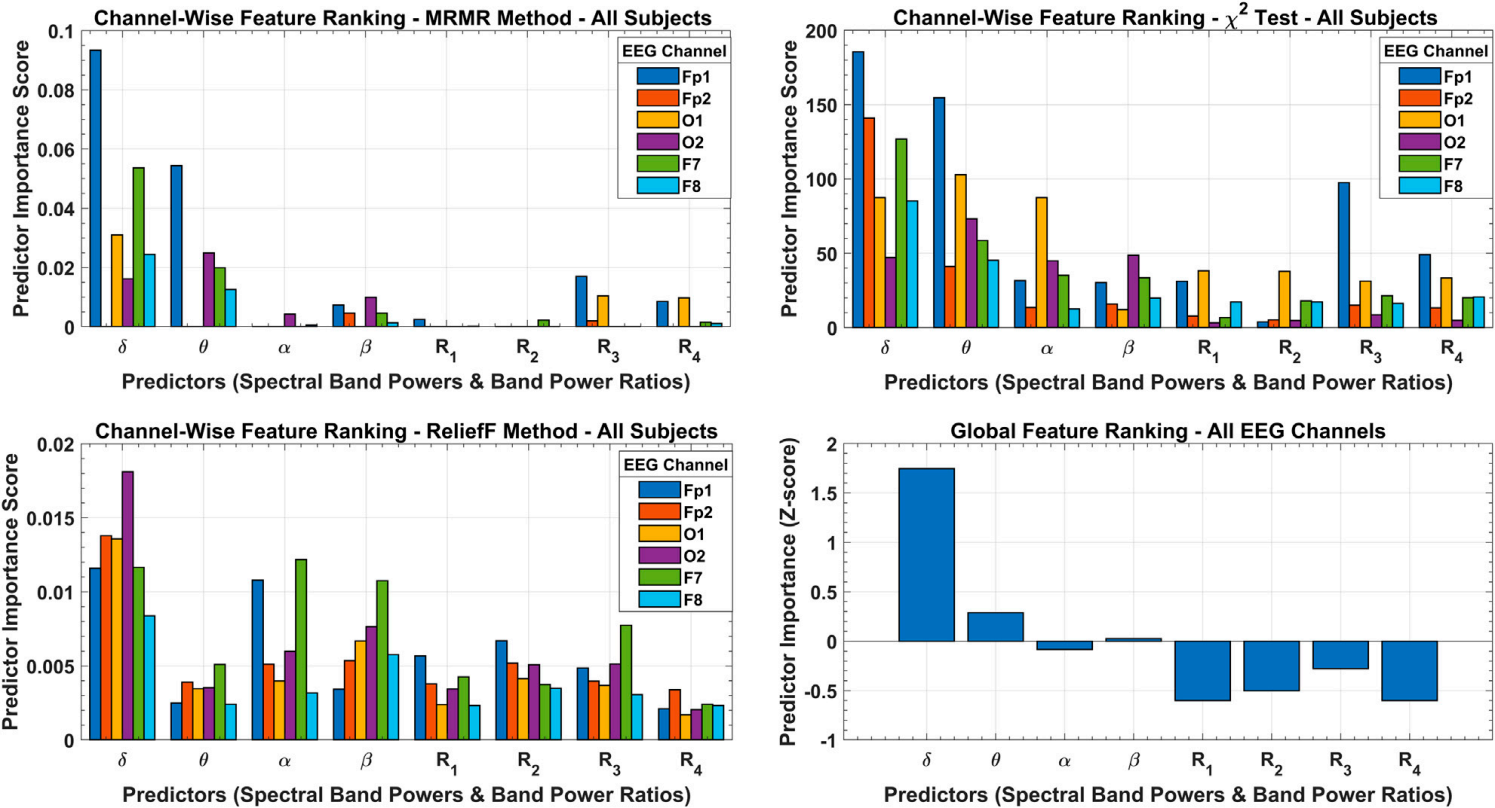
Channel-wise feature ranking with MRMR, chi-square, and ReliefF methods with predictor importance scores over all subjects’ data. The cumulative effect of all feature selection methods is shown with a global feature ranking scheme with predictor importance in terms of *Z*-score.

The results of the three feature selection approaches and their cumulative effect on global feature ranking are shown in Figure 8. According to the MRMR algorithm, only the δ and θ band powers are significant features with good predictor importance scores in all the channels except Fp2. Similarly, the $\chi^{2}$ test also rated δ and θ band powers with significantly higher importance scores but also rated other features with promising scores in contrast to MRMR, which almost subdued the other features. The ReliefF method rated δ , α , and β band powers as more important features in most of the channels, with θ band power and all ratio-based features having equal importance. The overall feature selection is somewhat ambiguous in these results, but Ζ -score-based global feature ranking made this selection very distinctive with efficient stacking of the three methods. All four band powers are important features according to the GPI score, with δ band power as the most significant feature. The other three band powers and $R_{3}$ BPR lie in the ± 0.3 standard deviation band of zero mean, which ascertains them as good candidates for the important feature set.

**FIGURE 9:**
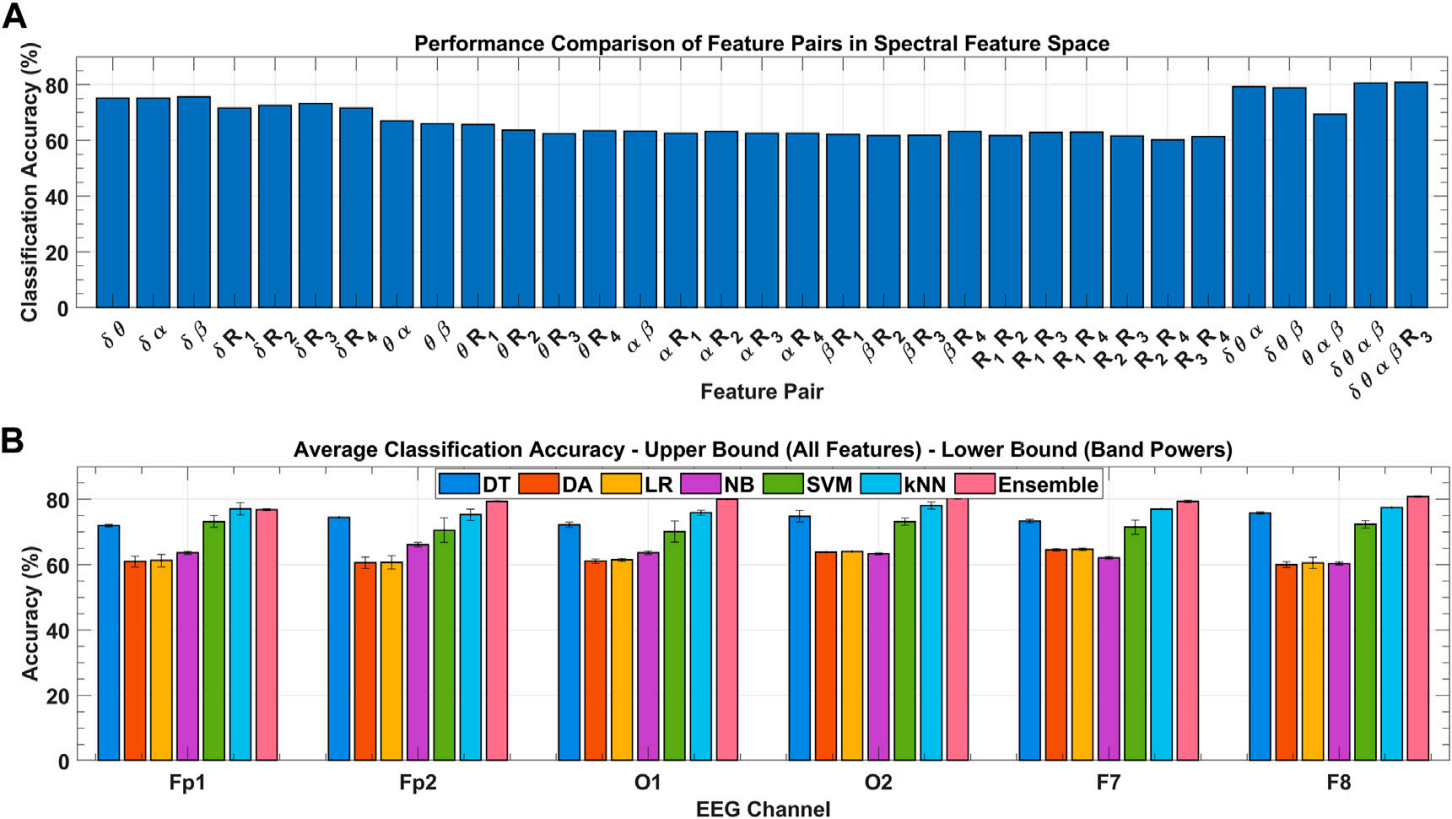
**(A)** Classification performance comparison among feature combinations of spectral feature space for the F8 channel with ensemble classifier over all subject’s data. **(B)** Classification performance comparison among various classifiers for the F8 channel over all subject’s data with variance bounds obtained with varying feature combinations.

To ensure the results of global feature ranking, the exhaustive embedded-type feature selection is performed based on the maximum relevance of the feature set to the response variable and is evaluated with the help of detection accuracy. Figure 9A shows the ensemble classification accuracy for all 2D feature pairs and higher-order combinations. All combinations of δ band power achieved higher classification accuracies (≥70%) than other feature combinations. This aspect validates the highest significance of the δ band power, as shown by its highest GPI score in Figure 8. The ternary combinations of δ with other band powers achieved almost 79% accuracy. The tertiary combination of all band powers achieved a classification accuracy of almost 82%, whereas the inclusion of the fifth significant feature ( $R_{3}$ BPR) did not further increase the accuracy significantly. The results of this approach agree with the global feature ranking. To minimize the feature extraction cost and yet achieve promising results, only the four most significant features (band powers) are selected.

### 3.3 Classification performance

Figure 9B shows the channel-wise average classification accuracies obtained using the seven classifiers under consideration over all subjects’ data. Error bars in each classifier were obtained with the use of different feature sets. Upper and lower bounds mark the accuracy obtained with the classification using; all eight spectral features, and the four band powers, respectively. In inter-classifier comparison, ensemble, k NN, SVM, and DT performed significantly well as compared to DA, LR, and NB classifiers for all the selected channels. The average classification accuracy with the ensemble is highest for all channels, followed by the k NN, except for Fp1, while DT and SVM also performed similarly after the two aforementioned classifiers. This variation in the classifier’s performance exists due to the use of different presets.

A similar classification process is also performed for all the subjects individually. Table 1 tabulates the subject-wise average classification accuracies obtained using all spectral features in all the selected channels. The best-performing variant of each classifier is also mentioned. The maximum accuracy achieved by a classifier with its preset is highlighted with bold values. Results are shown here for five subjects only, and statistical significance test results are discussed to conclude the entire population. All the classifiers achieved the highest accuracies in the F8 channel most of the time, with few exceptions of very insignificant differences, whereas the ensembles of bagged and boosted trees achieved the highest accuracies among all the classifiers in each EEG channel. There is a minor difference in the performance of ensemble, k NN, and DT for some of the subjects. The statistical significance test of 2-way ANOVA with replication is applied to all subjects’ data for validation of results. Test results show p<0.05 for both the inter-channel and inter-classifier comparisons, which effectively rejects the null hypothesis and indicates the significance of the obtained differences. So, conclusively, the bagged and boosted tree-based ensembles can be used for further binary classification of EEG signals in drowsy and alert brain states.

**TABLE 1:** Average classification accuracies (%) obtained with all spectral features, for all channels, and all the classifiers with best-performing presets (subjects 1–5).

Subject	Classifier (preset)	EEG channel					
		Fp1	Fp2	O1	O2	F7	F8
S1	DT (F-DT)	70.4	74.6	75.8	71.8	76.5	**78.3**
	DA (LDA)	**61.5**	61.4	58.2	57.1	61.3	56.0
					QDA		QDA
	LR	61.1	61.9	57.6	60.6	**63.1**	60.3
	NB (K-NB)	62.6	61.9	61.7	60.4	62.5	**64.0**
	SVM (FG-SVM)	61.8	63.1	66.1	**67.8**	67.6	67.6
	k NN (W- k NN)	70.4	79.4	78.1	73.8	74.6	**80.7**
		M- k NN		F- k NN			
	Ensemble (Ba-T)	75.1	76.1	79.7	79.3	80.6	**82.9**
S2	DT (M-DT)	69.0	71.6	75.4	80.3	79.7	**86.7**
		F-DT	C-DT	F-DT			
	DA (LDA)	67.5	67.1	67.5	67.1	67.5	**68.4**
	LR	67.5	66.5	67.3	66.9	67.1	**72.4**
	NB (K-NB)	67.7	66.7	67.5	**68.8**	66.4	73.7
		G-NB				G-NB	
	SVM (FG-SVM)	68.4	71.6	67.9	68.0	69.5	**78.2**
				MG-SVM			
	k NN (W- k NN)	71.8	77.4	76.9	77.1	**81.2**	79.3
	Ensemble (Ba-T)	70.1	73.7	77.1	81.6	82.7	**86.8**
					RBo-T	Bo-T	Bo-T
S3	DT (M-DT)	**81.4**	77.9	77.9	76.2	74.0	79.0
				F-DT		C-DT	
	DA (QDA)	71.3	68.8	**73.3**	71.5	70.3	67.5
					LDA		
	LR	68.6	64.8	**74.2**	70.4	72.4	71.0
	NB (K-NB)	72.9	71.3	73.8	69.8	**74.0**	71.5
	SVM (FG-SVM)	71.5	**76.8**	71.5	76.4	76.2	70.4
	k NN (W- k NN)	77.1	83.7	83.0	82.2	77.9	**84.9**
		F- k NN		F- k NN	F- k NN		
	Ensemble (Bo-T)	**81.9**	81.5	81.1	80.5	76.8	81.2
			Ba-T		Ba-T		
S4	DT (F-DT)	88.2	87.3	**89.0**	86.9	86.5	88.2
			C-DT			C-DT	
	DA (QDA)	**85.2**	82.7	81.9	84.0	**85.2**	81.4
				LDA			
	LR	84.0	82.3	81.9	83.5	**84.4**	82.7
	NB (K-NB)	87.3	**89.5**	81.4	83.1	87.3	85.7
				G-NB			
	SVM (FG-SVM)	**89.0**	87.3	85.2	86.1	**89.0**	87.3
		C-SVM				Q-SVM	Q-SVM
	k NN (W- k NN)	89.9	89.0	89.9	89.0	89.5	**91.1**
					F- k NN		F- k NN
	Ensemble (S- k NN)	89.0	91.1	90.3	90.7	90.3	**92.4**
		RBo-T		Ba-T	RBo-T		
S5	DT (M-DT)	75.2	80.5	85.2	84.2	79.7	**90.5**
			F-DT				
	DA (LDA)	72.0	71.4	81.6	75.1	71.6	**83.5**
					QDA		
	LR	72.0	71.8	84.1	76.7	71.5	**84.4**
	NB (K-NB)	73.2	73.8	83.4	75.9	77.0	**85.4**
					G-NB		
	SVM (FG-SVM)	72.3	76.1	83.7	78.5	77.8	**88.2**
	k NN (W- k NN)	73.3	78.7	84.9	82.7	79.4	**89.9**
				C- k NN			
	Ensemble (Ba-T)	79.6	81.1	87.8	85.9	81.6	**90.8**
				Bo-T			Bo-T

Note: F-DT: fine DT; M-DT: medium DT; C-DT: coarse DT; K-NB: kernel NB; G-NB: Gaussian NB; FG-SVM: fine Gaussian SVM; MG-SVM: medium Gaussian SVM; Q-SVM: quadratic SVM; C-SVM: cubic SVM; F- k NN: Fine k NN; M- k NN: medium k NN; W- k NN: weighted k NN; C- k NN: cosine k NN; Ba-T: bagged trees; Bo-T: boosted trees; RBo-T: RUSBoosted trees; S- k NN: subspace k NN. The highest value for each classifier is shown in bold. The statistical significance of the results is validated with replicated 2-way ANOVA ( p<0.05 ).

**FIGURE 10:**
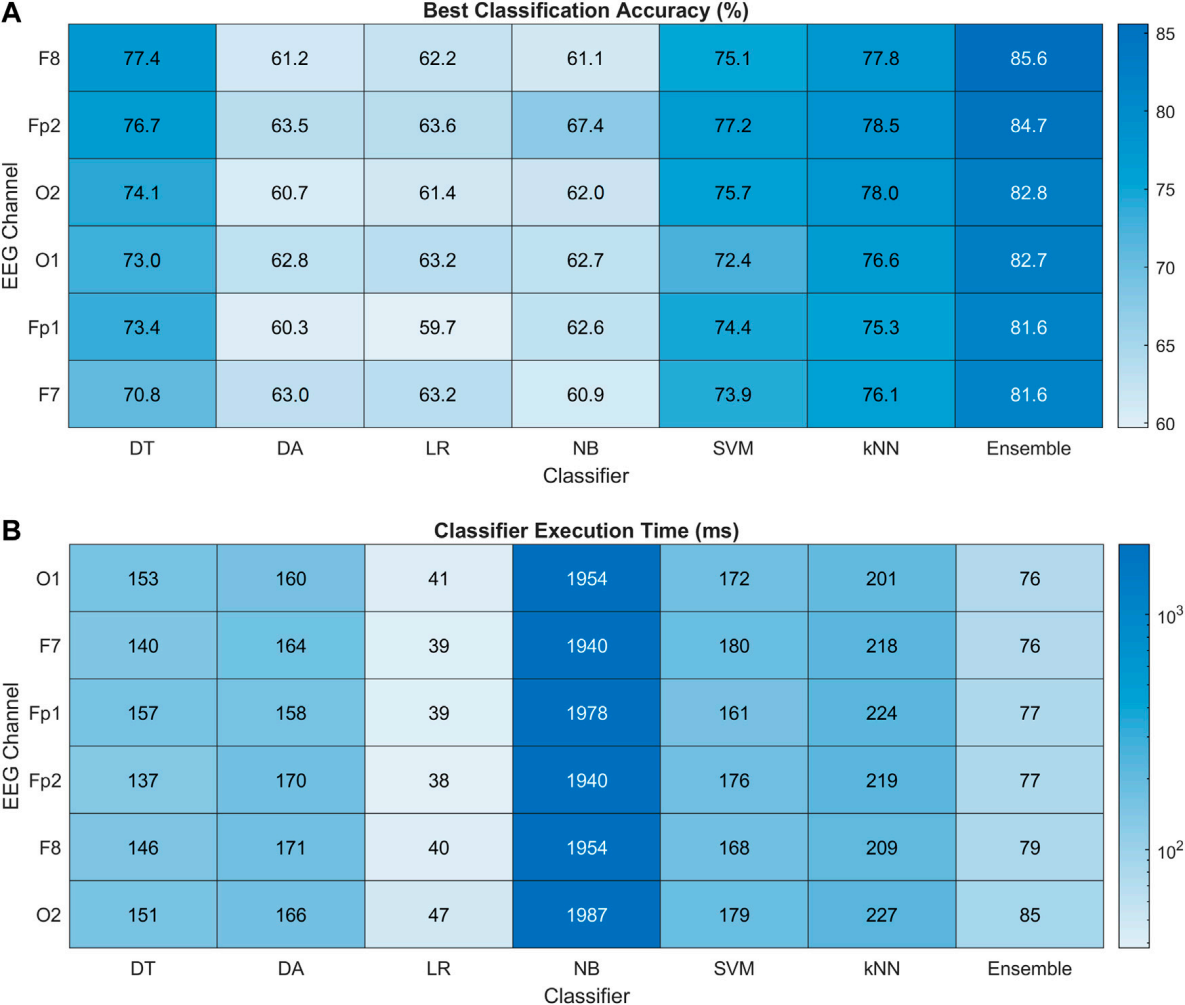
**(A)** Heat map chart for performance comparison with percentage accuracy of all the classification methods using δ,θ,α,β band powers only and channel ranking for the best classifier. **(B)** Classification computation time (ms) for all the classifiers mentioning their computational complexity for driving drowsiness assessment.

Figure 10A presents the heat map chart showing the best classification accuracies obtained for all the classifiers using the four most important spectral features (EEG band powers). These accuracies were obtained over five classification trials of each method in each channel. In addition, 10-fold cross-validation was used in each trial. All the classifiers achieved 60% and above classification accuracy, which is the minimum confidence threshold for BCI applications. Overall, the ensemble method achieved the best classification results among all the classifiers in all channels, with 85.6% highest accuracy obtained in the F8 channel of the right FC. The Fp2 is ranked second, followed by OC, and the left hemisphere is the last in this channel ranking. Figure 10B shows the execution time in milliseconds (ms) for all the classifiers. The LR classifier has the minimum execution time but achieved the minimum accuracies among all. In the best-performing classifiers, the ensemble model took the least execution time of 76 ms. The statistical significance of these results is validated with 2-way ANOVA tests. The inter-classifier comparison shows the statistically significant performance of the ensemble classifier ( p<0.05 ) for both the accuracy and execution time. In inter-channel comparison, significantly higher accuracies are achieved in F8 as compared to the others ( p<0.05 ), while there is an insignificant difference observed among them for classifier execution time ( p>0.05 ). These results recommend the F8 channel as COI for single channel-based drowsiness detection.

**FIGURE 11:**
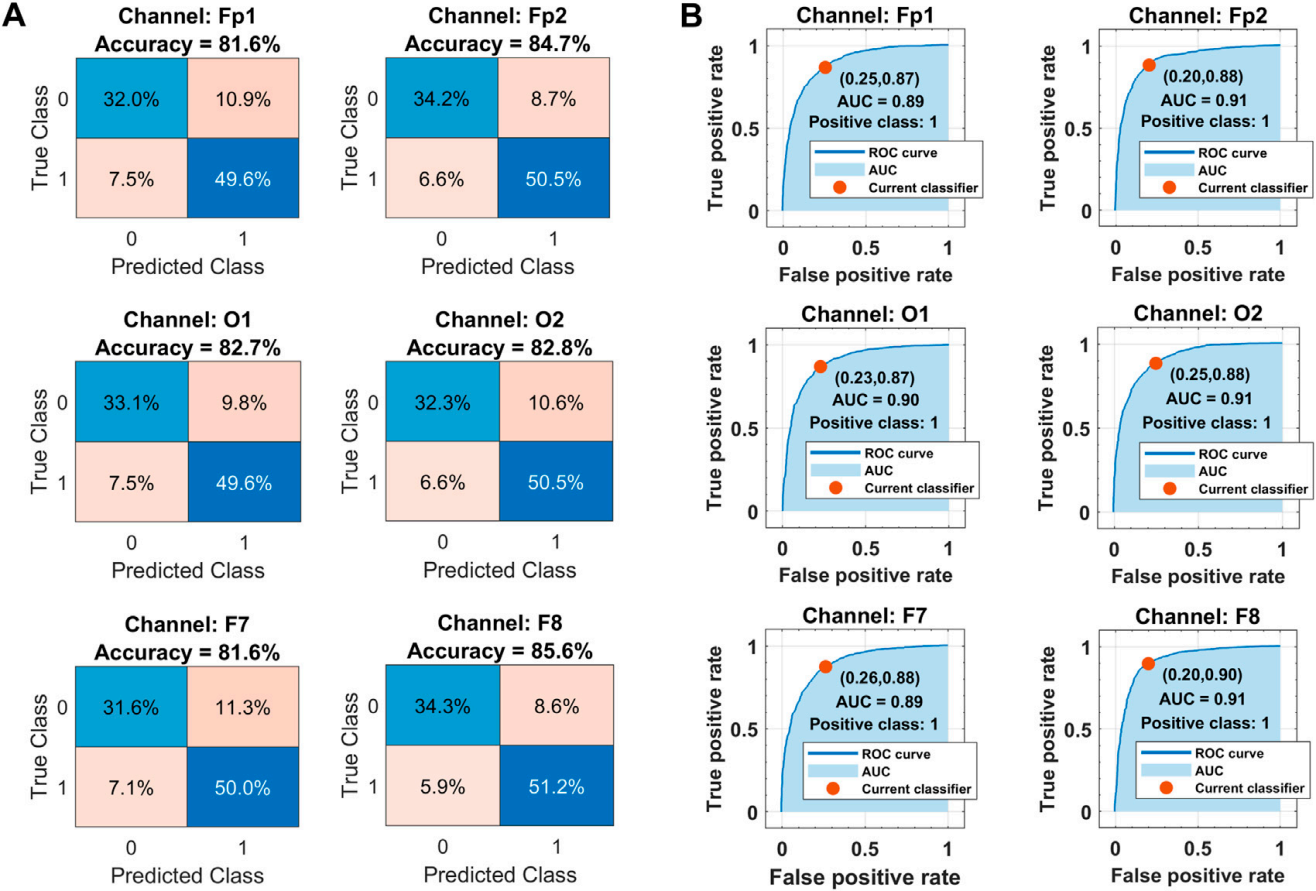
**(A)** Classification results in terms of confusion matrices. **(B)** Receiver operating characteristics curves over all subjects’ data in each selected channel with Bayesian optimization-based ensemble classifier. Class labels: 1 = drowsy, 0 = alert.

The corresponding confusion matrices and ROC curves for binary classification in each channel with the optimized ensemble are shown in Figure 11. Class labels “1” and “0” are assigned to drowsy and alert brain states, respectively. In Figure 11A, the diagonal and off-diagonal entries represent the correct and false classification percentages, respectively, with the normalized sample distribution in each class. The overall false detection rate is less in the range of 14%–18% against the higher accuracies. In Figure 11B, the ROC curves between sensitivity and fall-out represent the well-trained classifiers with 0.90 AUC, 88% sensitivity, and 23% fall-out on average for the drowsy class. The complete performance of the trained models is assessed with the help of other metrics too.

Table 2 shows the channel-wise results of confusion matrix-based performance assessment metrics obtained with the optimized ensemble classifier. Mainly, the optimized ensemble method is selected along with other hyperparameters’ tuning using Bayesian optimization. Multiple optimization sessions supported the confidence in using bagged trees to obtain the best classification results. All these reported results are obtained with bagged tree-based ensemble classifier and GPI ranked features, for all subjects’ data. The best results of all the metrics are obtained in the F8 channel, with 85.6% accuracy and precision, 89.7% sensitivity, 87.6% F_1_-score, 80% selectivity, 70.3% MCC, 70.2% kappa score, and 91% AUC. The single COI selection is validated with multiple paired Student’s t -tests between the results of F8 and all other channels. For multiple hypotheses testing ( m=5 ), Bonferroni correction is applied to obtain the new significant alpha threshold ( α=0.01 ). All t -statistics validated the statistical significance of F8 channel metrics ( p<0.01 ) compared to highly correlated metrics of other EEG channels ( r>.5 ).

**TABLE 2:** Confusion matrix-based performance evaluation metrics over all subjects’ data with globally ranked features and Bayesian optimization-based ensemble classifier.

Channel	Accuracy	Precision	Recall	F_1_-score	Specificity	MCC	Cohen’s kappa	AUC
Fp1	0.816	0.820	0.869	0.844	0.746	0.622	0.621	0.890
Fp2	0.847	0.853	0.884	0.868	0.797	0.686	0.686	**0.910**
O1	0.827	0.835	0.869	0.852	0.772	0.645	0.645	0.900
O2	0.828	0.827	0.884	0.854	0.753	0.647	0.645	**0.910**
F7	0.816	0.816	0.876	0.845	0.737	0.622	0.620	0.890
F8	**0.856**	**0.856**	**0.897**	**0.876**	**0.800**	**0.703**	**0.702**	**0.910**

Note: Statistical significance of the results is validated with paired t -test ( p<0.01 : Bonferroni corrected) for inter-channel comparison. The highest value of each metric is shown in bold.

## 4 Discussion

This EEG-based neurophysiology study is focused on achieving multiple objectives like feature selection to minimize the feature extraction cost at runtime, achieving higher drowsiness detection accuracy, and EEG channel selection to spatially localize the promising brain location for drowsiness detection. Reducing the number of electrodes up to one is achieved with channel selection. The single COI makes the pBCI hardware ergonomic by minimizing intrusion into normal driving tasks with accurate detection of passive brain activity. Various studies have investigated EEG-based drowsiness detection, but very few studies have worked upon all these objectives together to the best of the authors’ knowledge. The proposed pBCI scheme is compared with a few existing studies on various aspects, and the comparison results are summarized in Table 3. These works are similar and comparable as they have presented the offline driver drowsiness detection scheme based on physiological data collected during the lane-keeping task in a simulated driving environment.

**TABLE 3:** Detailed comparison of proposed drowsiness detection scheme with existing studies.

Reference	Subjects	Features	Classifier	Performance	Channels (location)
Awais et al. (2017)	11	Statistical temporal measures, spectral band powers, and HRV	SVM	Acc = 80.90%	2 (O2 and ECG)
Wei et al. (2018)	10	θ , α , and β spectral powers	LDA, k NN, SVM	Acc = 83.30%	4 (F7, F8, A1, and A2)
				AUC = 87.59%	
Choi et al. (2019)	8	MPSD	XGBoost	Acc = 78.51%	7 (Fp1, Fp2, T3, T4, O1, O2, and ECG)
				Acc-S = 91.32%	
				AUC = 86.80%	
				Sen = 78.51%	
				**Spec = 78.51%**	
Cui et al. (2021)	11	α spindles, spectral powers	CNN-LSTM	Acc = 72.97%	Single (Oz)
				Acc-S = 88.31%	
Shen et al. (2021)	11	δ , θ , α , and β spectral powers	MSSA-TN	Acc = 71.97%	30 (Multiple)
				Acc-S = 84.81%	
Cui et al. (2022)	11	α spindles and θ burst	CNN	Acc = 73.22%	Single (Oz)
				Acc-S = 88.25%	
Kim et al. (2022)	11	δ , θ , and α spectral powers	ResNet1D-18	Acc = 77.26%	32 (Multiple)
				Sen = 68.13%	
				F_1_-score = 62.66%	
Proposed	12	δ , θ , α , and β spectral powers	Ensemble (bagged trees)	**Acc = 85.50%**	Single (F8)
				**Acc-S = 92.40%**	
				**AUC = 90.20%**	
				**Sen = 88.00%**	
				Spec = 76.80%	
				**F**_**1**_ **-score = 85.70%**	

Note: CNN: convolution neural network; LSTM: long short-term memory; MSSA-TN: multi-source signal alignment *via* tensor network; MPSD: multitaper PSD; Acc: accuracy; Acc-S: subject-wise highest accuracy; Sen: sensitivity/recall; Spec: specificity. The highest value for each metric is shown in bold.

The feature extraction process significantly increases the processing time and computational cost during real-time classification tasks when a large number of features are involved. Predictor importance-based feature selection methods greatly optimize the feature extraction process by selecting only the promising and important features which are best representative of the response variable. Cui et al. (2021), Shen et al. (2021), Cui et al. (2022), and Kim et al. (2022) used DL models for feature extraction and classification, which incur higher computational costs, but they used spectral features, which are more effective and representative of physiological brain states. On the other hand, Awais et al. (2017), Wei et al. (2018), and Choi et al. (2019) used self-extracted spectral features and conventional ML classifiers for drowsiness detection, similar to this study. It is to be noted that the mentioned ML-based studies achieved higher classification accuracies and other metrics as compared to DL-based works. A possible reason for this difference is the use of hybrid physiological measures as Awais et al. (2017) and Choi et al. (2019) used ECG in addition to EEG measurements; Awais et al. (2017) also used statistical temporal features in their work. Both these studies used t -tests to find an important feature in the feature selection process. The proposed hybrid feature ranking scheme used in this study achieved the best results among all, with only four spectral features extracted from time-correlated spectral data and classified by ML classifiers with the least execution time. The minimum execution time of the overall scheme also resulted from reducing the number of channels required to be processed for activity classification. Shen et al. (2021) and Kim et al. (2022) used ≥30 EEG channels from multiple cortices, while other studies mentioned in Table 3 used fewer EEG channels (1–7) from selected cortices of the brain (PFC, FC, OC, TC, and mastoids). It is to be noted that all the studies have included OC channels (O1, O2, Oz) in their work, while FC channels (F7, F8) are among the selected channels in Wei et al. (2018), Choi et al. (2019) and this study. PFC channels (Fp1, Fp2) are also included in the analysis of this work and that of Choi et al. (2019). All the findings of the channel-wise performance of this study are positively correlated with all these comparative works. Awais et al. (2017), Cui et al. (2021), and Cui et al. (2022) effectively performed channel reduction up to a single COI for drowsiness detection in OC, whereas Awais et al. (2017) achieved higher accuracy due to hybrid physiology measures (EEG + ECG). In this study, these selected OC channels are promising and second in performance with comparatively better classification accuracy. However, our work has revealed significantly better results in FC channels which also performed better individually as compared to OC channels (Wei et al., 2018; Choi et al., 2019). These aspects support the confidence in our channel selection approach, which selected F8 as a single COI for drowsiness detection. In the comparison of performance assessment metrics, the proposed study achieved the best results with only a single COI and ML-based classifier. Overall accuracy, subject-wise highest accuracy, AUC, sensitivity, and F_1_-score are significantly higher than those in other works. The only exception is the specificity, which is higher in Choi et al. (2019) by 1.71%. The reason for the lower specificity of our work is the class imbalance in the dataset, as shown in Figure 11A. Additionally, the use of ECG (Choi et al., 2019) also contributed to the change in specificity results as HRV significantly varies in alert and drowsy states. The dataset of the proposed work contains 57.1% drowsy state and 42.9% alert state samples. This class imbalance of 7% induced bias in state detection probabilities, resulting in lower specificity. However, the performance results are still promising, with 76.8% and 88% correct detection rates of alert and drowsy states, respectively, with only a single EEG channel. As the number of subjects in our work is higher than in other studies, it may be a reason for achieving better results due to the availability of bigger training data. This aspect is promising as it provides more generalization effect and applicability for a bigger population.

The experimental procedure, collected dataset, used feature set, and time of drowsiness detection window have posed a few limitations in this study. As the dataset is collected during a simulated driving environment with offline data processing of the experiment, real driving experience may pose different issues for the proposed work. The participants belong to a certain age group with less frequent driving experience, so the proposed scheme should be adaptive for different classes of subjects. This study is focused on EEG spectral signatures only, so limited feature domains may have performance limitations. This work is effective for drowsiness detection in a 10-s time window, which should be reduced for earlier detection. In our ongoing research, neurophysiological data in controlled real driving experiments are being collected from comparatively aged subjects with higher commute frequencies as they are more likely to experience driving drowsiness. Furthermore, a new pBCI scheme is being developed with a significantly shorter time window for earlier drowsiness detection. The time domain characteristics of EEG, hybrid physiological measures, and DL-based classifiers are also being explored for further performance enhancement.

## 5 Conclusion

This study is aimed at designing a passive brain–computer interface (pBCI) scheme for driving drowsiness detection with minimum disruption to the driving task. As all the physiological states originate from the human brain, it is a promising location for earlier drowsiness detection in objective methods than that of behavioral and vehicular measures. The objective is to develop such a pBCI system that must be ergonomically less intrusive and easy to wear or place at the driver’s brain. It is possible if the pBCI scheme uses a fewer number of electrodes. To find out the single channel of interest is the main objective of this work.

The computational cost and algorithm execution time are minimized by introducing the feature ranking scheme in the feature extraction process. Multiple filter-type feature selection methods are ensembled with Ζ -score-based global feature ranking, and exhaustive embedded-type feature selection validated the feature ranking results. δ , θ , α , and β band powers were the most promising spectral features, with 85.6% best classification accuracy in a 10-s detection window. Multiple machine learning classifiers were tested for drowsiness classification in which bagged tree-based ensemble classifiers achieved the best results of confusion matrice-based performance assessment metrics. It reduced the execution time to 76 milliseconds, with the highest performance as compared to deep learning-based models. In inter-channel comparison, the best accuracy, precision, recall, F_1_-score, specificity, Matthews correlation coefficient, Cohen’s kappa, and area under the curve are achieved for the F8 channel of the frontal cortex in the right hemisphere of the brain. All the results are validated with statistical significance tests ( t -tests and ANOVA), which ascertained the F8 electrode position as COI having maximum spectral information gain and utility.

The presented single COI-based EEG neurophysiology scheme has a minimal ergonomic design which is easy to wear and less disruptive to normal driving tasks and practically detects the drowsiness correctly at an earlier stage to avoid life loss in vehicular driving scenarios. Improving the detection results with statistical temporal and spatiotemporal features in a smaller detection window or deep learning-based automatic feature extraction methods is the future direction of this study.

## Data Availability

The raw data supporting the conclusion of this article will be made available by the authors, without undue reservation.

## References

[B1] Abidi A. Ben Khalifa K. Ben Cheikh R. Valderrama Sakuyama C. A. Bedoui M. H. (2022). Automatic detection of drowsiness in EEG records based on machine learning approaches. Neural Process. Lett. 54, 5225–5249. 10.1007/s11063-022-10858-x

[B2] Aboalayon K. a. I. Faezipour M. Almuhammadi W. S. Moslehpour S. (2016). Sleep stage classification using EEG signal analysis: A comprehensive survey and new investigation. Entropy 18, 272. 10.3390/e18090272

[B3] Adão Martins N. R. Annaheim S. Spengler C. M. Rossi R. M. (2021). Fatigue monitoring through wearables: A state-of-the-art review. Front. physiology 12, 790292. 10.3389/fphys.2021.790292 PMC8715033 34975541

[B4] Ahn S. Nguyen T. Jang H. Kim J. G. Jun S. C. (2016). Exploring neuro-physiological correlates of drivers' mental fatigue caused by sleep deprivation using simultaneous EEG, ECG, and fNIRS data. Front. Hum. Neurosci. 10, 219. 10.3389/fnhum.2016.00219 27242483 PMC4865510

[B5] Akhtar T. Arif S. Mushtaq Z. Gilani S. O. Jamil M. Ayaz Y. (2022). “Ensemble-based effective diagnosis of thyroid disorder with various feature selection techniques,” in 2nd International Conference of Smart Systems and Emerging Technologies (SMARTTECH), Riyadh, Saudi Arabia, 09-11 May 2022 (IEEE), 14–19.

[B6] Akhtar T. Gilani S. O. Mushtaq Z. Arif S. Jamil M. Ayaz Y. (2021). Effective voting ensemble of homogenous ensembling with multiple attribute-selection approaches for improved identification of thyroid disorder. Electronics 10, 3026. 10.3390/electronics10233026

[B7] Akrout B. Mahdi W. (2021). A novel approach for driver fatigue detection based on visual characteristics analysis. J. Ambient Intell. Humaniz. Comput. 14, 527–552. 10.1007/s12652-021-03311-9

[B8] Ali I. Mushtaq Z. Arif S. Algarni A. D. Soliman N. F. El-Shafai W. (2023). Hyperspectral images-based crop classification scheme for agricultural remote sensing. Comput. Syst. Sci. Eng. 46, 303–319. 10.32604/csse.2023.034374

[B9] Alimardani M. Hiraki K. (2020). Passive brain-computer interfaces for enhanced human-robot interaction. Front. Robotics AI 7, 125. 10.3389/frobt.2020.00125 PMC7805996 33501291

[B10] Alotaiby T. El-Samie F. E. A. Alshebeili S. A. Ahmad I. (2015). A review of channel selection algorithms for EEG signal processing. EURASIP J. Adv. Signal Process. 2015, 66–21. 10.1186/s13634-015-0251-9

[B11] Arif S. Arif M. Munawar S. Ayaz Y. Khan M. J. Naseer N. (2021a). “EEG spectral comparison between occipital and prefrontal cortices for early detection of driver drowsiness,” in International Conference on Artificial Intelligence and Mechatronics Systems (AIMS), Bandung, Indonesia, 28-30 April 2021 (IEEE), 1–6.

[B12] Arif S. Khan M. J. Naseer N. Hong K. S. Sajid H. Ayaz Y. (2021b). Vector phase analysis approach for sleep stage classification: A functional near-infrared spectroscopy-based passive brain–computer interface. Front. Hum. Neurosci. 15, 658444. 10.3389/fnhum.2021.658444 33994983 PMC8121150

[B13] Awais M. Badruddin N. Drieberg M. (2017). A hybrid approach to detect driver drowsiness utilizing physiological signals to improve system performance and wearability. Sensors 17, 1991. 10.3390/s17091991 28858220 PMC5620623

[B14] Baiardi S. La Morgia C. Sciamanna L. Gerosa A. Cirignotta F. Mondini S. (2018). Is the Epworth Sleepiness Scale a useful tool for screening excessive daytime sleepiness in commercial drivers? Accid. Analysis Prev. 110, 187–189. 10.1016/j.aap.2017.10.008 29074223

[B15] Bamidele A. A. Kamardin K. Abd Aziz N. S. N. Sam S. M. Ahmed I. S. Azizan A. (2019). Non-intrusive driver drowsiness detection based on face and eye tracking. Int. J. Adv. Comput. Sci. Appl. 10, 775. 10.14569/ijacsa.2019.0100775

[B16] Belo J. Clerc M. Schön D. (2021). EEG-based auditory attention detection and its possible future applications for passive BCI. Front. Comput. Sci. 3, 661178. 10.3389/fcomp.2021.661178

[B17] Chaput J. P. Dutil C. Featherstone R. Ross R. Giangregorio L. Saunders T. J. (2020). Sleep timing, sleep consistency, and health in adults: A systematic review. Appl. Physiology, Nutr. Metabolism 45, S232–S247. 10.1139/apnm-2020-0032 33054339

[B18] Choi H. S. Min S. Kim S. Bae H. Yoon J. E. Hwang I. (2019). Learning-based instantaneous drowsiness detection using wired and wireless electroencephalography. IEEE Access 7, 146390–146402. 10.1109/access.2019.2946053

[B19] Collet C. Musicant O. (2019). Associating vehicles automation with drivers functional state assessment systems: A challenge for road safety in the future. Front. Hum. Neurosci. 13, 131. 10.3389/fnhum.2019.00131 31114489 PMC6503868

[B20] Croce P. Quercia A. Costa S. Zappasodi F. (2018). Circadian rhythms in fractal features of EEG signals. Front. physiology 9, 1567. 10.3389/fphys.2018.01567 PMC6240683 30483146

[B21] Cui J. Lan Z. Liu Y. Li R. Li F. Sourina O. (2022). A compact and interpretable convolutional neural network for cross-subject driver drowsiness detection from single-channel EEG. Methods 202, 173–184. 10.1016/j.ymeth.2021.04.017 33901644

[B22] Cui J. Lan Z. Zheng T. Liu Y. Sourina O. Wang L. (2021). “Subject-independent drowsiness recognition from single-channel EEG with an interpretable CNN-LSTM model,” in International Conference on Cyberworlds (CW), Caen, France, 28-30 September 2021 (IEEE), 201–208.

[B23] Diaz B. A. Hardstone R. Mansvelder H. D. Van Someren E. J. Linkenkaer-Hansen K. (2016). Resting-state subjective experience and EEG biomarkers are associated with sleep-onset latency. Front. Psychol. 7, 492. 10.3389/fpsyg.2016.00492 27148107 PMC4828461

[B24] Fathima S. Kore S. K. (2021). Formulation of the challenges in brain-computer interfaces as optimization problems—A review. Front. Neurosci. 14, 546656. 10.3389/fnins.2020.546656 33551716 PMC7859253

[B25] Hong K. S. Khan M. J. Hong M. J. (2018). Feature extraction and classification methods for hybrid fNIRS-EEG brain-computer interfaces. Front. Hum. Neurosci. 12, 246. 10.3389/fnhum.2018.00246 30002623 PMC6032997

[B26] Hong K. S. Khan M. J. (2017). Hybrid brain–computer interface techniques for improved classification accuracy and increased number of commands: A review. Front. neurorobotics 35, 35. 10.3389/fnbot.2017.00035 PMC5522881 28790910

[B27] Hu J. (2017a). Automated detection of driver fatigue based on AdaBoost classifier with EEG signals. Front. Comput. Neurosci. 11, 72. 10.3389/fncom.2017.00072 28824409 PMC5540979

[B28] Hu J. (2017b). Comparison of different features and classifiers for driver fatigue detection based on a single EEG channel. Comput. Math. methods Med. 2017, 5109530. 10.1155/2017/5109530 28255330 PMC5307247

[B29] Huang C. S. Lin C. L. Ko L. W. Liu S. Y. Su T. P. Lin C. T. (2014). Knowledge-based identification of sleep stages based on two forehead electroencephalogram channels. Front. Neurosci. 8, 263. 10.3389/fnins.2014.00263 25237291 PMC4154530

[B30] Jabbar R. Shinoy M. Kharbeche M. Al-Khalifa K. Krichen M. Barkaoui K. (2020). “Driver drowsiness detection model using convolutional neural networks techniques for android application,” in IEEE International Conference on Informatics, IoT, and Enabling Technologies (ICIoT), Doha, Qatar, 02-05 February 2020 (IEEE), 237–242.

[B31] Kartsch V. J. Benatti S. Schiavone P. D. Rossi D. Benini L. (2018). A sensor fusion approach for drowsiness detection in wearable ultra-low-power systems. Inf. Fusion 43, 66–76. 10.1016/j.inffus.2017.11.005

[B32] Khan H. Naseer N. Yazidi A. Eide P. K. Hassan H. W. Mirtaheri P. (2021). Analysis of human gait using hybrid EEG-fNIRS-based BCI system: A review. Front. Hum. Neurosci. 14, 613254. 10.3389/fnhum.2020.613254 33568979 PMC7868344

[B33] Khan R. A. Naseer N. Qureshi N. K. Noori F. M. Nazeer H. Khan M. U. (2018). fNIRS-based Neurorobotic Interface for gait rehabilitation. J. neuroengineering rehabilitation 15, 7–17. 10.1186/s12984-018-0346-2 PMC5800280 29402310

[B34] Khan R. A. Naseer N. Saleem S. Qureshi N. K. Noori F. M. Khan M. J. (2020). Cortical tasks-based optimal filter selection: An fNIRS study. J. Healthc. Eng. 2020, 1–15. 10.1155/2020/9152369

[B35] Kim D. Y. Han D. K. Jeong J. H. Lee S. W. (2022). "EEG-Based driver drowsiness classification via calibration-free framework with domain generalization,” in IEEE International Conference on Systems, Man, and Cybernetics (SMC), Prague, Czech Republic, 09-12 October 2022 (IEEE), 2293–2298.

[B36] Larocco J. Le M. D. Paeng D. G. (2020). A systemic review of available low-cost EEG headsets used for drowsiness detection. Front. neuroinformatics 42, 553352. 10.3389/fninf.2020.553352 PMC7593569 33178004

[B37] Li G. Chung W. Y. (2022). Electroencephalogram-based approaches for driver drowsiness detection and management: A review. Sensors 22, 1100. 10.3390/s22031100 35161844 PMC8840041

[B38] Li X. Song D. Zhang P. Zhang Y. Hou Y. Hu B. (2018). Exploring EEG features in cross-subject emotion recognition. Front. Neurosci. 12, 162. 10.3389/fnins.2018.00162 29615853 PMC5867345

[B39] Liu S. Shen J. Li Y. Wang J. Wang J. Xu J. (2021). EEG power spectral analysis of abnormal cortical activations during REM/NREM sleep in obstructive sleep apnea. Front. Neurology 12, 643855. 10.3389/fneur.2021.643855 PMC7953149 33716946

[B40] Min J. Wang P. Hu J. (2017). Driver fatigue detection through multiple entropy fusion analysis in an EEG-based system. PLoS one 12, e0188756. 10.1371/journal.pone.0188756 29220351 PMC5722287

[B41] Naseer N. Noori F. M. Qureshi N. K. Hong K. S. (2016). Determining optimal feature-combination for LDA classification of functional near-infrared spectroscopy signals in brain-computer interface application. Front. Hum. Neurosci. 10, 237. 10.3389/fnhum.2016.00237 27252637 PMC4879140

[B42] Nazeer H. Naseer N. Khan R. A. Noori F. M. Qureshi N. K. Khan U. S. (2020a). Enhancing classification accuracy of fNIRS-BCI using features acquired from vector-based phase analysis. J. Neural Eng. 17, 056025. 10.1088/1741-2552/abb417 33055382

[B43] Nazeer H. Naseer N. Mehboob A. Khan M. J. Khan R. A. Khan U. S. (2020b). Enhancing classification performance of fNIRS-BCI by identifying cortically active channels using the z-score method. Sensors 20, 6995. 10.3390/s20236995 33297516 PMC7730208

[B44] Poursadeghiyan M. Mazloumi A. Saraji G. N. Niknezhad A. Akbarzadeh A. Ebrahimi M. H. (2017). Determination the levels of subjective and observer rating of drowsiness and their associations with facial dynamic changes. Iran. J. public health 46, 93–102. 28451534 PMC5401941

[B45] Pudjihartono N. Fadason T. Kempa-Liehr A. W. O'sullivan J. M. (2022). A review of feature selection methods for machine learning-based disease risk prediction. Front. Bioinforma. 2, 927312. 10.3389/fbinf.2022.927312 PMC9580915 36304293

[B46] Quercia A. Zappasodi F. Committeri G. Ferrara M. (2018). Local use-dependent sleep in wakefulness links performance errors to learning. Front. Hum. Neurosci. 12, 122. 10.3389/fnhum.2018.00122 29666574 PMC5891895

[B47] Qureshi N. K. Naseer N. Noori F. M. Nazeer H. Khan R. A. Saleem S. (2017). Enhancing classification performance of functional near-infrared spectroscopy-brain–computer interface using adaptive estimation of general linear model coefficients. Front. neurorobotics 33, 33. 10.3389/fnbot.2017.00033 PMC5512010 28769781

[B48] Qureshi N. K. Noori F. M. Abdullah A. Naseer N. (2016). “Comparison of classification performance for fNIRS-BCI system,” in 2nd International Conference on Robotics and Artificial Intelligence (ICRAI), Rawalpindi, Pakistan, 01-02 November 2016 (IEEE), 54–57.

[B49] Radüntz T. (2017). Dual frequency head maps: A new method for indexing mental workload continuously during execution of cognitive tasks. Front. physiology 8, 1019. 10.3389/fphys.2017.01019 PMC5727053 29276490

[B50] Ruffini G. Ibañez D. Castellano M. Dubreuil-Vall L. Soria-Frisch A. Postuma R. (2019). Deep learning with EEG spectrograms in rapid eye movement behavior disorder. Front. neurology 10, 806. 10.3389/fneur.2019.00806 PMC6683849 31417485

[B51] Rundo F. Conoci S. Spampinato C. Leotta R. Trenta F. Battiato S. (2021). Deep neuro-vision embedded architecture for safety assessment in perceptive advanced driver assistance systems: The pedestrian tracking system use-case. Front. neuroinformatics 15, 667008. 10.3389/fninf.2021.667008 PMC8361480 34393746

[B52] Sasaki M. Iversen J. Callan D. E. (2019). Music improvisation is characterized by increase EEG spectral power in prefrontal and perceptual motor cortical sources and can be reliably classified from non-improvisatory performance. Front. Hum. Neurosci. 13, 435. 10.3389/fnhum.2019.00435 31920594 PMC6915035

[B53] Shen M. Zou B. Li X. Zheng Y. Li L. Zhang L. (2021). Multi-source signal alignment and efficient multi-dimensional feature classification in the application of EEG-based subject-independent drowsiness detection. Biomed. Signal Process. Control 70, 103023. 10.1016/j.bspc.2021.103023

[B54] Singh D. Singh B. (2020). Investigating the impact of data normalization on classification performance. Appl. Soft Comput. 97, 105524. 10.1016/j.asoc.2019.105524

[B55] Tanveer M. A. Khan M. J. Qureshi M. J. Naseer N. Hong K. S. (2019). Enhanced drowsiness detection using deep learning: An fNIRS study. IEEE access 7, 137920–137929. 10.1109/access.2019.2942838

[B56] Wei C. S. Wang Y. T. Lin C. T. Jung T. P. (2018). Toward drowsiness detection using non-hair-bearing EEG-based brain-computer interfaces. IEEE Trans. neural Syst. rehabilitation Eng. 26, 400–406. 10.1109/TNSRE.2018.2790359 29432111

[B57] Zhou D. Li X. (2020). Epilepsy EEG signal classification algorithm based on improved RBF. Front. Neurosci. 14, 606. 10.3389/fnins.2020.00606 32655355 PMC7324866

